# Folate metabolism in tumor-associated macrophages drives immunosuppressive function to promote tumor growth

**DOI:** 10.64898/2026.09.29.755523

**Published:** 2026-10-02

**Authors:** Matthew P. Zimmerman, Vasyl Zhabotynsky, Haiyang Wang, Emily K. Cox, Wan Lin Chong, Alex G. Bastian, Andrew S. Kennedy, Brian P. Fay, Amy G. Reynolds, Anna Ebacher, Jeremy A. Meier, Katherine Vietor, Khalilah E. Taylor, Pristine C. Onuoha, Siddharth Maruvada, Katie E. Hurst, Christopher Wong, Carlton W. Anderson, Nancy E. Thomas, Rihe Liu, Kirsten L. Bryant, Albert S. Baldwin, David W. Ollila, Jenny P-Y Ting, Blake R. Rushing, Susan J. Sumner, Sergey A. Krupenko, Willy Hugo, Stergios J. Moschos, Jessica E. Thaxton, Brian C. Miller

**Affiliations:** 1. Department of Cell Biology and Physiology, University of North Carolina at Chapel Hill, Chapel Hill, NC 27514; 2. Lineberger Comprehensive Cancer Center, University of North Carolina at Chapel Hill, Chapel Hill, NC 27514; 3. Department of Pharmacology, University of North Carolina at Chapel Hill, Chapel Hill, NC 27514; 4. Department of Biology, High Point University, High Point, NC 27268; 5. Department of Microbiology and Immunology, University of North Carolina at Chapel Hill, Chapel Hill, NC 27514; 6. Carbone Cancer Center, University of Wisconsin at Madison, Madison, WI and Cancer Biology Graduate Program, University of Wisconsin at Madison, Madison, WI, USA; 7. Department of Medicine, Division of Hematology, University of North Carolina at Chapel Hill, Chapel Hill, NC 27514; 8. North Carolina State University, Raleigh, North Carolina, 27695; 9. Department of Dermatology, University of North Carolina at Chapel Hill, Chapel Hill, NC 27514; 10. Division of Chemical Biology and Medicinal Chemistry, Eshelman School of Pharmacy, University of North Carolina at Chapel Hill, NC 27514; 11. Department of Surgery, University of North Carolina at Chapel Hill, Chapel Hill, NC, 27514; 12. Department of Genetics and Molecular Biology, University of North Carolina at Chapel Hill, Chapel Hill, NC 27514; 13. Department of Nutrition, University of North Carolina at Chapel Hill, Chapel Hill, NC, 27514; 14. Nutrition Research Institute, University of North Carolina at Chapel Hill,, Kannapolis, NC 28081; 15. Department of Medicine, Division of Dermatology, University of California, Los Angeles, Los Angeles, CA 90095; 16. Center for Gastrointestinal Biology and Disease, University of North Carolina at Chapel Hill, Chapel Hill, NC 27514; 17. Department of Medicine, Division of Medical Oncology, University of North Carolina at Chapel Hill, Chapel Hill, NC 27514

## Abstract

Tumor-associated macrophages (TAMs) promote tumor growth, inhibit effector lymphocytes, and induce resistance to immune checkpoint inhibitors (ICI). Therapies designed to deplete TAMs have had little success in the clinic, and strategies aimed to repolarize immunosuppressive TAMs to pro-inflammatory states have struggled with systemic toxicity. We have identified that the combination of low folate in the tumor microenvironment and TAM dependency on the high affinity folate receptor beta (FRβ) for folate uptake results in a unique metabolic dependency of TAMs necessary for their immunosuppressive function. FRβ (encoded by *Folr2*) is uniquely expressed on myeloid cells and upregulated on TAMs. FRβ+ TAMs exhibit an immunosuppressive phenotype in melanoma and are associated with worse clinical outcomes and resistance to ICI in melanoma patients. We generated a novel *Folr2*^*−/−*^ mouse model to study why TAMs express a unique folate receptor and showed that tumor growth was slowed in the absence of *Folr2* in a T-cell dependent manner. We observed a dramatic repolarization of *Folr2*^*−/−*^ TAMs *in vivo* to pro-inflammatory states, resulting in increased cytotoxic T cell and NK cell infiltration into tumors. Importantly, we show that folate is low in the tumor microenvironment by performing metabolomics on melanoma tumors and adjacent normal tissue from warm autopsy patient specimens, and that we could increase tumor growth in *Folr2*^*−/−*^ mice by increasing serum folate to supraphysiologic concentrations with a high folate diet. Integrated metabolomics and transcriptomic analysis demonstrated that *Folr2*^*−/−*^ macrophages have impaired folate uptake and 1C metabolism-based reduction of oxidized glutathione in low folate conditions, resulting in increased mitochondrial reactive oxygen species (ROS) in *Folr2*^*−/−*^ cells. Excess ROS results in leakage of mitochondrial DNA into the cytoplasm, activating cGAS-STING signaling and promoting pro-inflammatory macrophage polarization through TBK1 and NF-kB. Together, our data demonstrate that TAM expression of FRβ promotes their immunosuppressive functions by maintaining folate uptake in the low folate tumor microenvironment, suggesting that FRβ is a novel metabolic checkpoint on TAMs.

## Introduction

Despite the success of immune checkpoint inhibitors (ICI), such as antibodies targeting PD-1/PD-L1, to treat a variety of cancers, the majority of patients do not respond. Tumor-associated macrophages (TAMs) can inhibit T cell function and the efficacy of ICI, and are associated with worse clinical outcomes for patients.^1,2^ However, TAMs are functionally and transcriptionally heterogenous, depending on cancer type, developmental origin, and the local microenvironmental signals. Therapeutic strategies to deplete or repolarize TAMs into anti-tumor effectors have had little clinical success, likely because of poor selectivity for TAMs over resident tissue macrophages (RTMs) and our limited understanding of the pathways driving TAM polarization.

Metabolism and metabolic byproducts are important for macrophage polarization, particularly in the tumor microenvironment (TME).^3^ One carbon (1C) metabolism is a central metabolic pathway required by all cells and is upregulated during both pro-inflammatory and immunosuppressive macrophage polarization.^4–7^ Folate is a required co-enzyme for 1C metabolism, and antifolate drugs that inhibit 1C metabolism are commonly used chemotherapies and immunosuppressants (e.g. methotrexate, pemetrexed, 5-FU).^8^ In macrophages, folate deprivation or treatment with antifolates increases pro-inflammatory cytokine production, although the mechanisms are unknown.^9–11^

1C metabolism is critical for *de novo* nucleotide synthesis, generation of one carbon units for cellular methylation reactions, and control of reactive oxygen species (ROS) within cells.^8^ When cells are deficient in folate, ROS levels increase.^12^ 1C metabolism supports NADPH production, which is required to reduce the cellular antioxidant glutathione, and also regulates mitochondrial function, a significant source of ROS production.^13–16^ ROS are important signaling molecules in macrophages, driving either pro- or anti-tumor functions, depending on the intracellular location of ROS production, type of ROS, and duration and levels of ROS.^17,18^ ROS induce immunosuppressive macrophage polarization through activation of Keap1-Nrf2 and Jak-Stat6 signaling.^19,20^ Conversely, ROS promote proinflammatory polarization through activation of NF-kB and HIF-1⍺ signaling.^21,22^

Myeloid cells, particularly macrophages, uniquely express a high affinity folate receptor, FRβ (gene: *Folr2*), one of 4 functional folate carriers found in mice (FRα, FRβ, RFC, PCFT). RFC (Reduced Folate Carrier, gene: *Slc19a1*) is expressed by all mammalian cells, whereas PCFT (Proton Coupled Folate Transporter, gene: *Slc46a1*) is involved in folate uptake in low pH environments (e.g. gut lumen, choroid plexus).^23^ Unlike these low affinity folate transporters, FRβ has about 1000x higher affinity for folates, particularly oxidized folates such as folic acid.^23^ However, because it is GPI anchored, folates bound to FRβ must enter the cell through endocytosis, instead of direct membrane transport, resulting in lower capacity uptake than the folate transporters.^24^ Unlike knockout mice of the other folate transporters, *Folr2*^*−/−*^ mice are viable and do not have neural tube defects.^23,25^ FRβ is upregulated on activated macrophages and TAMs in the TME, where it has been associated with both immunosuppressive and pro-inflammatory TAM phenotypes.^26–30^ Recently, it has been suggested that FRβ may have functions independent of folate uptake – regulating macrophage migration, serving as a direct checkpoint of T cell activity, promoting inflammasome activation, and sustaining TGF-β signaling.^31–34^ Multiple groups have developed FRβ-targeted therapeutics to deplete FRβ^+^ TAMs or deliver specific drugs to these cells,^35–40^ however we still do not understand how FRβ regulates TAM biology nor the role of FRβ^+^ TAMs in mediating resistance to immune checkpoint inhibitors.

In this study we demonstrate that FRβ is expressed on TAMs with an immunosuppressive phenotype in human and mouse melanoma and is associated with a poor prognosis for melanoma patients. To interrogate the functions of FRβ on TAMs, we generated a novel C57BL/6J *Folr2*^*−/−*^ mouse model by CRISPR-Cas9. Tumors implanted into *Folr2*^*−/−*^ mice grew more slowly due to CD8^+^ T cell mediated tumor control. Loss of *Folr2* promoted pro-inflammatory TAM polarization *in vivo*, resulting in activation of innate and adaptive lymphocytes. We demonstrate that *Folr2*^*−/−*^ bone marrow derived macrophages (BMDMs) grown in low folate media replicate the transcriptional and functional phenotypes of *Folr2*^*−/−*^ TAMs and can control tumor growth when injected into melanoma tumors. Proving that these phenotypes are due to deficient folate uptake by *Folr2*^*−/−*^ cells, we found that feeding *Folr2*^*−/−*^ mice a high folate diet or growing *Folr2*^*−/−*^ BMDMs in high folate media reverses pro-inflammatory macrophage polarization and tumor growth phenotypes. To dissect the mechanisms by which loss of *Folr2* repolarizes macrophage biology we integrated metabolomics and transcriptional analysis of *Folr2*^*−/−*^ and wild-type BMDMs, demonstrating that FRβ-mediated folate uptake is required for mitochondrial function and control of ROS production. In the absence of FRβ, mitochondrial DNA leaks into the cytoplasm, activating cGAS-STING and NF-κB signaling pathways to induce macrophage polarization. Finally, we demonstrate that loss of FRβ synergizes with anti-PD-1. Together, our data demonstrate that TAMs require FRβ for folate uptake in the low folate TME to prevent mitochondrial-induced pro-inflammatory polarization.

## Results

### FRβ is expressed by immunosuppressive TAMs and is associated with poor patient outcomes for patients with melanoma.

FRβ expression has been associated with both immunosuppressive and pro-inflammatory macrophages, depending on the specific tumor type or inflammatory context.^26–30^ To evaluate the role of FRβ+ TAMs in melanoma, we first integrated and then analyzed two published single-cell RNA-seq (scRNA-seq) datasets from melanoma patients.^41,42^ We identified a robust population of *FOLR2*^+^ TAMs that co-expressed the pro-tumor TAM markers *MRC1* (CD206), *CD209*, and *CD163* (Fig. 1A). Next, we obtained fresh biopsies of 6 tumors from 4 patients with advanced melanoma undergoing resection for Lifileucel (TIL) therapy, demonstrating that about 60% of TAMs on average expressed FRβ in advanced tumors (Fig. 1B, Supp. Fig. 1A). FRβ+ TAMs had higher expression of the immunosuppressive markers CD163, CD206, and PD-L1 versus FRβ- TAMs, supporting our previous scRNAseq analysis findings (Fig. 1C, Supp. Fig. 1B). To determine if FRβ+ TAMs are significant for melanoma patient prognosis, we stratified patient survival in the melanoma (SKCM) TCGA dataset (n = 432 patients, most tumors collected before ICI approval) based on the relative expression of *FOLR2* and *CD8A*. Patients with a high ratio of *FOLR2/CD8A* – suggesting a relative abundance of FRβ^+^ TAMs over CD8^+^ T cells – had significantly worse overall survival (Fig. 1D). Survival analysis using a Cox model adjusting for *CD8A*, age, sex, and weight also showed that higher expression of *FOLR2* was associated with worse survival (Fig. 1E). Together, these results suggest that FRβ+ TAMs have an immunosuppressive phenotype and function in melanoma tumors.

Next, we interrogated the expression of FRβ by TAMs in a variety of mouse tumor models. We observed between 20% - 80% of TAMs expressed FRβ, depending on the specific model (Fig. 1F, Supp. Fig. 1C). Importantly, this expression was specific to TAMs and not observed on any other cell type in the tumor microenvironment (TME). To determine how specific FRβ is for TAMs compared with resident tissue macrophages, we performed flow cytometry on leukocytes found in healthy C57BL/6J mouse kidneys, lung, spleens, and liver (Fig. 1F, Supp. Fig. 1D). Only Kupffer cells had robust expression of FRβ, although other cell types in the liver did have low-level expression (Fig. 1E). Recent work suggests that FRβ may be important for development of liver fibrosis.^34^ We conclude that only TAMs and Kupffer cells express high levels of FRβ. Similarly, in human data from the Human Protein Atlas, *Folr2* transcript and FRβ protein were only robustly detected in macrophages (Supp. Fig. 1E, 1F).^43,44^ Finally, we evaluated expression of macrophage polarization markers in murine FRβ+ TAMs, and found that across tumor models FRβ+ TAMs had higher expression of Arginase 1 and CD206 and lower expression of MHC-II, consistent with a more immunosuppressive phenotype (Fig. 1G–J, Supp. Fig. G).

### FRβ is required for immunosuppressive TAM polarization *in vivo*.

To evaluate FRβ’s mechanistic role in TAM polarization and anti-tumor immunity, we generated *Folr2*^*−/−*^ mice on the C57BL/6J background using CRISPR-Cas9-based genetic engineering to remove all coding DNA sequences (Fig. 2A). Unlike previously reported *Folr2*^*−/−*^ mice, our mice had no evidence of systemic autoimmunity, with unchanged total body and slightly reduced spleen weights (Supp. Fig. 2A,2B).^32^ We also observed no birth defects or deviations from expected pup numbers. We grew bone-marrow derived macrophages (BMDMs) *in vitro* or sorted TAMs from tumors implanted into wild-type and *Folr2*^*−/−*^ mice for flow cytometry and RNA-seq. BMDMs and TAMs isolated from *Folr2*^*−/−*^ mice completely lacked *Folr2* transcript and FRβ protein expression (Fig. 2B, 2C).

To determine if *Folr2* is important for TAM function, we subcutaneously implanted a variety of syngeneic tumor cell lines into *Folr2*^*−/−*^ and control mice. Tumor growth was significantly reduced across a variety of tumor models, with a 60% reduction in parental B16F10 tumors and 45% reduction in immunogenic B16F10-OVA tumors (Fig. 2D). We next isolated CD45^+^ TILs from B16F10 tumors and performed single-cell RNA-sequencing to interrogate how loss of *Folr2* altered the TME (Supp. Fig. 2C-E). First, we confirmed that the *Folr2* transcript is only detected in macrophages in WT mice (Supp. Fig. 2F, 2G). Subclustering on myeloid and dendritic cells, we observed a dramatic repolarization of the myeloid compartment. The TME of *Folr2*^*−/−*^ mice were depleted of interstitial (*Msr1*, *Vcan*, *Spp1*) and hypoxic-niche (*Vegfa*, *Hif1a*, *Egln3*) TAMs and enriched in ISG^hi^ (*Mx1*, *Isg15*, *Cxcl10*) and efferocytic (*Axl*, *Mertk*) TAMs, suggesting a more inflamed TME (Fig. 2E–G, Supp. Fig. 2H, 2I). Indeed, differential gene expression analysis of TAMs from *Folr2*^*−/−*^ and control mice revealed enrichment of interferon-stimulated and antigen presentation signatures in *Folr2*^*−/−*^ TAMs versus hypoxia and TGFβ signatures in control TAMs (Fig. 2H). These findings were orthogonally confirmed by enrichment of pro-inflammatory gene expression pathways in our bulk RNAseq analysis of TAMs sorted from B16F10-OVA tumors (Supp. Fig. 2J). We also observed an increase in interferon–stimulated neutrophils in *Folr2*^*−/−*^ tumors, even though neutrophils did not express *Folr2*, consistent with these cells responding to a more pro–inflammatory TME (Supp. Fig. 2K–M). Pseudotime analysis of neutrophils demonstrated that these cells branched early and progressed down a distinct differentiation pathway in *Folr2*^*−/−*^ mice, likely driven by interferon signaling (Supp. Fig. 2N). Flow cytometry analysis of TAMs from B16F10 tumors implanted into *Folr2*^*−/−*^ and control mice revealed an increase in CD86^+^MHC-II^+^ TAMs and decrease in CD206 and Arg1 expression, consistent with an inflammatory phenotype (Fig. 2I). To functionally interrogate these TAMs, we sorted myeloid cells from B16F10 tumors for *ex vivo* cytokine production and found that *Folr2*^*−/−*^ TAMs made more pro-inflammatory IL-12/23p40 (Fig. 2J). Finally, to directly test if *Folr2*^*−/−*^ macrophages control tumor growth, we cultured BMDMs from wild-type or *Folr2*^*−/−*^ mice and implanted them into B16F10-OVA tumors on day 9 post tumor implantation. *Folr2*^*−/−*^ BMDMs significantly reduced tumor growth compared with control BMDMs (Fig. 2K). Together, these results demonstrate that FRβ promotes TAM immunosuppressive polarization in the TME.

### FRβ expression on TAMs inhibits immune effector cells.

We next asked how FRβ+ TAMs impact immune effector cells. To evaluate the role of FRβ+ TAMs in melanoma, we first performed spatial transcriptomics of liver, lung, lymph node, and brain melanoma metastases isolated from 8 patients who donated their bodies to the UNC warm autopsy program.^45,46^ Because our Visium data does not have single-cell resolution, instead capturing up to 10 cells per cluster, we generated cell-type signatures from scRNA-seq data and used these to deconvolute cell populations within Visium clusters. We identified many clusters containing *FOLR2*^+^ TAMs (red clusters, Fig. 3A). *FOLR2*^+^ TAM clusters frequently contained exhausted CD8^+^ T cells, but had moderate to poor spatial overlap with other T cell activation states (Fig. 3B). This analysis supports the hypothesis that FRβ^+^ TAMs inhibit T cell activity.

To directly interrogate this hypothesis, we returned to our mouse model. The B16F10-OVA cell line was engineered to express the strong xenoantigen, ovalbumin, that drives a robust CD8^+^ T cell response against the tumor.^47^ To determine if CD8^+^ T cells are required for control of B16F10-OVA tumors in *Folr2*^−/−^ mice, we depleted >98% of CD8^+^ T cells from the TME using anti-CD8α or isotype control antibodies (Supp Fig. 3A). In the absence of CD8^+^ T cells, there was no longer a difference in tumor size between *Folr2*^*−/−*^ and control mice, suggesting that the effects of *Folr2* depletion are due to improved CD8^+^ T cell tumor control (Fig. 3C). Indeed, flow cytometry confirmed an increase in Granzyme B expressing CD8^+^ T cells in *Folr2*^*−/−*^ mice (Fig. 3D). However, we also observed tumor control in cancer models without strong xenoantigens, such as B16F10. We did not detect major changes in the numbers or activation states of CD8^+^ T cells or CD4^+^ T cells in B16F10 tumors by flow cytometry (data not shown). However, NK cell numbers were increased in B16F10 tumors in *Folr2*^*−/−*^ mice compared with control mice, suggesting activation of other innate lymphocytes to control B16F10 tumor growth (Fig. 3E). Returning to our scRNA-seq data from B16F10 tumors, we noticed an increase in innate-like lymphocytes such as yδ T cells and NK cells (Fig. 3F–H, Supp. Fig. 3B). These data support the hypothesis that FRβ expression on TAMs is required for inhibition of multiple immune effectors, depending on the specific tumor context.

To further interrogate the crosstalk between myeloid cells and lymphocytes in these tumors, we performed CellChat, an algorithm that predicts potential interactions between cell populations using known ligand-receptor pairs.^48^ We observed an increase in communication between the efferocytic, ISG^hi^, and *Lyve1*^+^ (*Lyve1, Selenop, Cd163*) TAMs with many other cell clusters in *Folr2*^*−/−*^ mice, suggesting that deletion of *Folr2* increases communication between these populations and other immune cells (Fig. 3I, Supp. Fig. 3C). The most highly upregulated pathways identified in these TAM populations were MHC-I, CD86, and chemokines, suggesting upregulation of antigen presentation and immune cell recruitment by *Folr2*^*−/−*^ TAMs (Fig. 3J). Of note, *Folr2*^*−/−*^ TAMs had a decrease in TGFβ signaling, consistent with the overall decrease in this pathway we previously observed (Fig. 2H). Globally, signaling from TAMs with an immunosuppressive phenotype (interstitial, hypoxic-niche, and *Trem2*^+^) was decreased in the absence of *Folr2*^*−/−*^, and on the receiving side, we observed an increase in signaling to cycling lymphocytes and effector and exhausted CD8^+^ T cells in *Folr2*^*−/−*^ mice (Fig. 3I).

### Low intracellular folate is responsible for the pro-inflammatory phenotype of *Folr2*^*−/−*^ macrophages.

To study how FRβ expression alters macrophage biology, we next developed an *in vitro* system to model the TME. Given that prior work has suggested that IL-4 + M-CSF treatment of BMDMs *in vitro* best replicates the differentiation state of TAMs *in vivo*,^49^ we elected to use IL-4 polarization for our *in vitro* culture system. In addition to cytokines, we wanted to appropriately model the folate concentrations found in the TME in our *in vitro* cultures. We isolated tumor interstitial fluid (TIF) from mouse B16F10 tumors or human fresh melanoma biopsies obtained from patients undergoing tumor resection for Lifeulicel TIL therapy and measured folate concentration by ELISA. In both mouse and human tumors, TIF folate concentrations ranged from 5 – 10ng/mL, 100-fold less than the 1000ng/mL found in standard tissue culture media (Fig. 4A). In addition, we performed metabolomics of melanoma metastases and adjacent healthy tissue isolated from warm autopsy patients.^45,46^ These studies revealed a decrease in intratumoral folates compared with normal adjacent tissue, suggesting that melanoma tumors are depleted of folates (Fig. 4B). We therefore cultured wild-type and *Folr2*^*−/−*^ BMDMs for 6–7 days in folate-free media supplemented with dialyzed FBS and known concentrations of folic acid for all *in vitro* experiments. At physiologic folate concentrations (10ng/mL) we detected a significant decrease in the intracellular folate concentration in *Folr2*^*−/−*^ BMDMs that was nearly the same as cells grown in media without folate (Fig. 4C). However, the differences in folate were rescued in high folate media, suggesting other folate transporters expressed by *Folr2*^*−/−*^ macrophages could potentially offset decreased uptake mediated by loss of FRβ. Using our bulk RNA-seq data generated from wild-type BMDMs and TAMs, we found that macrophages express *Folr2* at > 30x higher levels than either *Slc19a1* or *Slca46a1*, the two low-affinity folate transporters (Fig. 4D, Supp. Fig. 4A). There was no compensatory increase in other folate transporter expression when *Folr2* was deleted (Supp. Fig. 4B). We hypothesize that at sufficiently high folate concentrations, the low affinity folate transporters can compensate for the loss of *Folr2* to maintain intracellular folate levels, but *Folr2* is critically required in low folate environments.

We first noticed a decrease in *Folr2*^*−/−*^ BMDM numbers in low folate media due to an increase in cell death (Supp. Fig. 4C–E). To assess BMDM function we therefore normalized for cell number or performed single-cell readouts. Similar to our TAM scRNA–seq and bulk RNA-seq data, we observed an enrichment of pro-inflammatory signatures in BMDMs (Fig. 4E). In fact, there was excellent overlap between signatures enriched in *Folr2*^*−/−*^ TAMs and IL-4 polarized *Folr2*^*−/−*^ BMDMs, suggesting shared biology mediated by loss of FRβ in both *in vitro* and *in vivo* settings and supporting our use of M(IL-4) BMDMs for functional studies (Supp. Fig. 4F). Interestingly, expression of pro-inflammatory genes could be reversed by culturing *Folr2*^*−/−*^ BMDMs in high folate media (Fig. 4F). Combined with our intracellular folate concentration results (Fig. 4C), these data support the hypothesis that high folate media can rescue the pro-inflammatory switch observed with loss of FRβ.

To assess how *Folr2* deletion affected macrophage polarization, we evaluated various metrics of macrophage phenotype and function. Both CD301 and Arginase 1 expression were decreased in *Folr2*^*−/−*^ BMDMs, but expression was rescued by high folate media (Fig. 4G). Similarly, production of pro-inflammatory cytokines IL-12/23p40, TNFα, IL-1β, and IL-6 were increased in *Folr2*^*−/−*^ BMDMs and reversed by high folate media (Fig. 4H). We did not detect transcriptional expression of the immunosuppressive cytokine IL-10 in either wild-type or *Folr2*^*−/−*^ BMDMs, nor was there any change in IL-10 expression in TAMs *ex vivo* (data not shown). We next tested if *Folr2*^*−/−*^ BMDMs could inhibit T cell activation by co-culturing M(IL-4) *Folr2*^*−/−*^ and wild-type BMDMs with CTV-labeled CD8^+^ T cells stimulated with anti-CD3/CD28 (Fig. 4I). Whereas wild-type BMDMs robustly inhibited T cell proliferation, cytokine production, and activation, *Folr2*^*−/−*^ BMDMs cultured in low folate media did not (Fig. 4J). *Folr2*^*−/−*^ BMDMs cultured in high folate media reversed this phenotype (Fig. 4J, Supp. Fig. 4G). Finally, we fed mice a high folate diet (8.6mg/kg, compared with 0.2mg/kg low folate diets) that increased their serum folate levels by 8–10 fold (Supp. Fig. 4H). We observed no differences in red or white blood cell parameters on the different diets, suggesting that mice tolerated the different diets without obvious evidence of folate deficiency (Supp. Fig. 4I). Tumor growth in wild–type mice was unchanged when comparing mice on low and high folate diets, suggesting that the different diets did not directly impact tumor cell growth (Supp. Fig. 4J). However, congruent with the findings from our high folate media rescue experiments conducted *in vitro*, *Folr2*^*−/−*^ mice fed a high folate diet no longer controlled tumor growth better than control mice, arguing that sufficient levels of folate could rescue the loss of FRβ on TAMs (Fig. 4K). Taken together, our *in vitro* and *in vivo* rescue data strongly support the conclusion that the primary function of FRβ on TAMs is to maintain folate uptake in low folate environments.

### Folate uptake is required to prevent oxidative stress in macrophages.

To interrogate the mechanisms by which low folate uptake induces pro-inflammatory polarization, we performed untargeted metabolomics on M(IL-4) *Folr2*^*−/−*^ and wild-type BMDMs cultured in low or high folate media and integrated this analysis with our transcriptional data (Fig. 5A, 5B, Supp. Fig. 5A–5C). Component analysis revealed dramatic metabolomic and transcriptomic differences between *Folr2*^*−/−*^ and wild-type BMDMs cultured in low folate media, but very little differences when the cells were cultured in high folate media (Fig. 5A, 5B, Supp. Fig. 5B, 5C). In fact, the largest component for both datasets captured differences based on the previously-observed gradients of intracellular folate concentrations (Fig. 5A, 5B, 4C).

We focused our analysis on *Folr2*^*−/−*^ and wild-type cells cultured in low folate media. Transcriptionally, *Folr2*^*−/−*^ BMDMs were enriched for pathways of reactive oxygen species and DNA damage, whereas wild-type cells were enriched for oxidative phosphorylation and fatty acid biosynthesis (Fig. 5C). Metabolomics pathway enrichment identified expected changes in one carbon (1C) metabolism and downstream metabolic pathways, such as pyrimidine, glutathione, and glycine/serine metabolism (Fig. 5D). Reassuringly, folate species and derivatives (5-methyltetrahydrofolate, folic acid, and pterine) were the most highly depleted metabolites in *Folr2*^*−/−*^ BMDMs in low folate media, confirming our previous ELISA data (Fig. 4C, Fig. 5E, 5F, Supp. Fig. 5D). Of note, all metabolomic differences observed between wild-type and *Folr2*^−/−^ macrophages were completely lost in high folate media (Fig. 5F, Supp. Fig. 5E). Looking across significantly altered metabolites (FDR < 0.1) in M(IL-4) BMDMs cultured in low folate media, a clear pattern emerged: N-formylmethionine was reduced in *Folr2*^*−/−*^ BMDMs, suggesting an inhibition of mitochondrial translation,^50^ with a subsequent increase in fatty acids (propionylcarnitine, tetradecanoylcarnitine, and oleoylcarnitine) consistent with impaired fatty acid metabolism and mitochondrial function. There was also evidence of increased ROS in *Folr2*^*−/−*^ BMDMs, with increased oxidized glutathione and cholesterol-5α,6α-epoxide (an oxidized form of cholesterol) and a concomitant decrease in reduced glutathione (Fig. 5E, 5G, Supp. Fig. 5D). 1C metabolism is a critical cellular source of both both NADPH for maintenance of cellular redox state and NADH for oxidative phosphorylation. Perhaps unsurprisingly, NADH and NAPDH were also decreased in *Folr2*^*−/−*^ cells (Fig. 5H). Transcriptionally, we observed a dramatic decrease in mitochondrial 1C enzymes in *Folr2*^*−/−*^ BMDMs, consistent with our GSEA analysis (Fig. 5I, Fig. 5C, Supp. Fig. 5F). Together with our transcriptional data demonstrating an enrichment in ROS and NRF2 signaling pathways in *Folr2*^*−/−*^ BMDMs (Fig. 5E), this integrative analysis suggests that *Folr2*^*−/−*^ macrophages are experiencing oxidative stress due to impaired mitochondrial function in physiologic folate conditions.

To test this hypothesis, we measured ROS within cells using CellRox, a global cellular oxidative stress indicator, or MitoSox, a mitochondria-localized superoxide probe. We observed no difference in CellRox staining between wild-type and *Folr2*^*−/−*^ BMDMs in low folate media, but a significant increase in MitoSox staining in *Folr2*^*−/−*^ BMDMs that could be rescued by high folate media (Fig. 5J, 5K), suggesting that mitochondria ROS accumulation is the source of the oxidative stress observed in *Folr2*^*−/−*^ BMDMs.

ROS have a complicated role in macrophages, important for both pro-inflammatory and immunosuppressive polarization.^17,18^ To test if excess ROS induces pro-inflammatory functions in *Folr2*^*−/−*^ BMDMs, we treated the cells with excess glutathione (GSH) or n-acetylcysteine (NAC) to decrease excess ROS. MitoSox staining was reduced by both GSH and NAC, demonstrating the activity of these antioxidants in our system (Supp. Fig. 5G). Treatment with either antioxidant increased expression of the immunosuppressive markers CD301 and Arg1 and decreased production of IL-12/23p40, TNFα, and IL-1β by *Folr2*^*−/−*^ BMDMs (Fig. 5L–N). Furthermore, pre-treatment of *Folr2*^*−/−*^ BMDMs with NAC prior to co-culture with activated T cells increased the T cell suppressive capacity of *Folr2*^*−/−*^ BMDMs (Fig. 5O). ROS can activate the inflammasome, and prior work in the THP-1 leukemia cell line suggests that FRβ activates the inflammasome independent of folate.^33^ To determine if there was evidence of inflammasome activation or inhibition in *Folr2*^*−/−*^ BMDMs, we measured ASC puncta formation and IL-1β production. We did not observe changes in ASC puncta formation nor inflammasome-mediated IL-1β cytokine production in *Folr2*^*−/−*^ BMDMs (Supp. Fig. 5H, 5I). Together, these functional studies demonstrate that excess ROS produced by *Folr2*^*−/−*^ BMDMs are critical for the pro-inflammatory functions of *Folr2*^*−/−*^ BMDMs independent of the inflammasome.

### Folate uptake by FRβ is required to prevent mitochondrial dysfunction.

Given our data implicating excess mitochondrial ROS in the pro-inflammatory polarization of *Folr2*^*−/−*^ BMDMs, we wanted to understand if mitochondria were dysfunctional in *Folr2*^*−/−*^ macrophages. Transcriptionally we observed a decrease in oxidative phosphorylation and electron transport chain signatures in *Folr2*^*−/−*^ TAMs and BMDMs (Fig. 5C, Fig. 6A, Supp. Fig. 6A). We measured mitochondrial membrane potential and mass using the dyes TMRM and Mitotracker Green, respectively. We observed no difference in mitochondrial mass between wild-type and *Folr2*^*−/−*^ BMDMs, but we did find a decrease in mitochondrial membrane potential in *Folr2*^*−/−*^ BMDMs, a hallmark of mitochondrial dysfunction (Fig. 6B). We next directly assessed mitochondrial oxygen consumption using Seahorse. *Folr2*^*−/−*^ BMDMs had decreased basal respiratory capacity and mitochondrial ATP production as well as increased basal extracellular acidification, resulting in a decreased OCR/ECAR ratio (Fig. 6C, Supp. Fig. 6B). These data suggest that *Folr2*^*−/−*^ BMDMs have dysfunctional mitochondria and a metabolic shift towards glycolysis, and closely resemble mitochondrial function in cells lacking SHMT2, a key enzyme needed for mitochondrial folate metabolism.^50^ Next, we performed transmission electron microscopy of wild-type and *Folr2*^*−/−*^ BMDMs, revealing an increase in phagolysosomes in *Folr2*^*−/−*^ BMDMs. In addition, mitochondria in *Folr2*^*−/−*^ BMDMs had disorganized cristae and frequently localized within lysosomes in *Folr2*^*−/−*^ BMDMs, suggesting mitochondrial degradation (Fig. 6D, Supp. Fig. 6C). We confirmed that *Folr2*^*−/−*^ BMDMs had an increase in mitochondrial degradation by immunofluorescence staining, revealing many more colocalized mitochondria (TOM20+) and lysosomes (LAMP1+) in *Folr2*^*−/−*^ BMDMs (Fig. 6E). However, we did not observe significant changes in LC3 conversion by immunoblot, arguing against a significant increase in total autophagic flux in *Folr2*^*−/−*^ BMDMs (Supp. Fig. 6D).

Mitochondria are key signaling hubs in macrophages. Although ROS can directly activate pathways important for pro-inflammatory phenotype, such as NF-κB and HIF-1α, damaged mitochondria can also release mitochondrial DNA, activating cGAS-STING. Activated STING trafficks from the endoplasmic reticuluum to the golgi apparatus and recruits cytosolic kinase TBK1, which potentiates NF-κB- and IRF3-mediated signaling to induce pro-inflammatory cytokine production. We interrogated activation of this pathway by phospho-immunoblot, demonstrating that *Folr2*^*−/−*^ BMDMs have increased NF-κB p65 phosphorylation in low folate media that is rescued by high folate media (Fig. 6F). Similarly, TBK1 phosphorylation increased and total STING levels decreased, consistent with activation of STING and TBK1 (Fig. 6G). These results support the hypothesis that leakage of mitochondrial DNA is a driver of macrophage polarization in folate-deficient *Folr2*^*−/−*^ BMDMs.

Excess ROS production by mitochondria results in oligomerization of VDAC1 channels in the mitochondrial membrane, promoting mitochondrial DNA release. We next asked if inhibition of mitochondrial ROS by the antioxidant MitoTEMPO, which localizes to the mitochondria and neutralizes superoxide, could reverse the phenotype of *Folr2*^*−/−*^ BMDMs. Indeed, treatment of *Folr2*^*−/−*^ BMDMs with MitoTEMPO decreased pro-inflammatory cytokine production and increased the suppressive capacity of these macrophages (Fig. 6H). To test if mitochondrial DNA leak contributes to *Folr2*^*−/−*^ macrophage pro-inflammatory function, we treated *Folr2*^*−/−*^ BMDMs with VBIT-4, a small molecular inhibitor of VDAC1 oligomerization that inhibits mitochondrial DNA release.^51^ Treatment with VBIT-4 reduced inflammatory cytokine production by *Folr2*^*−/−*^ BMDMs (Fig. 6J). In summary, our data support that folate uptake by FRβ in low folate conditions, such as the TME, is critical to prevent mitochondrial dysfunction, ROS production, and mitochondrial DNA release. In the absence of FRβ, macrophages become polarized due to activation of STING-mediated signaling by mitochondrial DNA.

### FRβ^+^ TAMs inhibit the efficacy of immune checkpoint inhibitors.

ICI have become the backbone of therapy for patients with advanced melanoma. Immunosuppressive TAMs are associated with resistance to ICI in melanoma.^1,2^ To determine if FRβ^+^ TAMs induce resistance, we first analyzed a published single-cell dataset of 32 melanoma patients who received ICI.^42^ We identified an increase in *FOLR2*^+^ TAMs in non-responders, and very few *FOLR2*^+^ TAMs in responding patients (Fig. 7A). Next, we asked if anti-PD-1 immunotherapy is more effective in *Folr2*^*−/−*^ mice. We implanted B16F10-OVA tumor cells, which are responsive to anti-PD-1, into wild-type or *Folr2*^*−/−*^ mice and treated them with anti-PD-1 or isotype control antibodies. We dosed the mice late with anti-PD-1 to minimize responsiveness in wild-type mice and allow us to assess synergy between anti-PD-1 and loss of *Folr2*. We observed a dramatic decrease in tumor growth in *Folr2*^*−/−*^ mice treated with anti-PD-1, but minimal effects of anti-PD-1 in wild-type mice (Fig. 7B). We then harvested tumors for flow cytometry to determine how the combination of anti-PD-1 and loss of *Folr2* impacted immune infiltrates. We observed an increase in immune effector cells (NK cells and CD8^+^ T cells) and increase in Granzyme B^+^ CD8^+^ T cells in anti-PD-1 treated compared with isotype-treated *Folr2*^*−/−*^ mice (Fig. 7C, 7D). Together, these results suggest that repolarizing TAMs into pro-inflammatory cells by loss of *Folr2* further improves the efficacy of anti-PD-1 therapy.

## Discussion

Immunosuppressive tumor-associated macrophages promote tumor growth, inhibit immune effector cells, and confer resistance to ICI for melanoma patients, highlighting the need for new therapeutic strategies to target TAMs. Using a novel *Folr2*^*−/−*^ mouse model, we demonstrate that FRβ promotes immunosuppressive TAM polarization and function in the TME. We identify that FRβ^+^ TAMs inhibit T cells and innate lymphocytes to enable tumor growth. FRβ is required for folate uptake by TAMs in low folate environments, and using fresh human melanoma resections we demonstrate that the melanoma TME is a low folate environment relative to surrounding normal tissue. Through integrative metabolic and transcriptional analysis we found that in the absence of folate uptake by FRβ, macrophages accumulate dysfunctional mitochondria which produce excess ROS and leak mitochondrial DNA, activating the cGAS-STING pathway to promote pro-inflammatory macrophage polarization (Fig. 7E). Finally, we show that FRβ^+^ TAMs are associated with resistance to ICI in melanoma patients, and loss of *Folr2* synergizes with anti-PD-1 immunotherapy in mice to control tumor growth. Together, these results demonstrate that FRβ is a novel metabolic checkpoint on TAMs that is a critical determinant of inflammatory phenotype and function.

Importantly, we can reverse the pro-inflammatory functions of *Folr2*^*−/−*^ BMDMs *in vitro* and tumor control in *Folr2*^*−/−*^ mice by supplementing the cells or mice with excess folic acid, demonstrating that these phenotypes are dependent on folate. Furthermore, our mechanistic work demonstrating that inhibition of ROS or mitochondrial DNA release also impairs the pro-inflammatory polarization of *Folr2*^*−/−*^ BMDMs further supports our folate-dependent mechanism. Although our data do not support non-folate roles of FRβ that have been postulated in the literature,^31–34^ we cannot rule out that FRβ has other functions in TAMs in addition to folate uptake. Interestingly, prior work has suggested that not all FRβ expressed by cells can bind folate, suggesting there may be an inactive confirmation of this protein.^35^ The relevance of this is unknown, but may explain non-folate mediated roles of FRβ observed in other contexts.

ROS activation of the Keap1-Nrf2 axis is thought to be critical for immunosuppressive TAM polarization.^19^ Conversely, acute production of ROS after LPS stimulation is required for optimal pro-inflammatory polarization of macrophages.^21,22^ ROS are complicated signaling molecules, with many species having very short half-lives. As a result, the location and type of ROS production, duration of ROS signaling, and local concentration of anti-oxidants all factor into the downstream effects of ROS on macrophage signaling and function.^17,18^ We identified that excess mitochondrial ROS are responsible for the phenotype of *Folr2*^*−/−*^ BMDMs. We hypothesize that impaired control of ROS is due to decreased mitochondrial 1C metabolism, which is essential for generating reducing power.^13,15^ There may also be a feed-forward loop occurring in our *Folr2*^*−/−*^ macrophages, whereas excess ROS damages the electron transport chain, resulting in increased superoxide production.^52^ We propose that excess ROS in mitochondria induces oligomerization of VDAC1 channels, allowing mitochondrial DNA escape to activate cGAS-STING, resulting in pro-inflammatory polarization of *Folr2*^*−/−*^ macrophages.

Although we believe that our BMDM model system recapitulates the key findings of *Folr2*^*−/−*^ TAMs *in vivo* given the similar functional, phenotypic, and transcriptional changes observed, there are also key differences. First, we have not observed a decrease in *Folr2*^*−/−*^ TAM numbers *in vivo*, despite the increase in cell death of *Folr2*^*−/−*^ BMDMs *in vitro*. This may be due to rapid influx of new monocytes *in vivo* to replace dying *Folr2*^*−/−*^ TAMs. Alternatively, we used the more stable folic acid *in vitro*, whereas the predominant form of folate *in vivo* is the reduced 5-methyltetrahydrofolate (5-MTHF). RFC does not bind well to folic acid, so it is likely that the *Folr2*^*−/−*^ BMDMs are more folate deficient than *Folr2*^*−/−*^ TAMs.^53^ Finally, although our *in vivo* single-cell RNA-seq data give us a detailed view of the immune infiltrates in *Folr2*^*−/−*^ mice, they do not allow us to determine which effects are directly due to loss of *Folr2* and which are secondary effects from downstream activation of other immune cells (e.g. increased IFNу production by activated T cells). For example, there are striking changes in neutrophils resulting in differentiation towards an interferon-sensing transcriptional state (Supp. Fig. 2K–M). We do not detect *Folr2* expression in tumor–associated neutrophils (Supp. Fig. 2G), nor is the protein FRβ found by flow cytometry on these cells (Fig. 1F), suggesting that these changes are due to secondary effects. One of these enriched states in *Folr2*^*−/−*^ TAMs are efferocytotic TAMs that express high levels of the receptors Tyro3, Axl, and MerTK, which promote immunosuppressive TAM functions. We hypothesize that these efferocytic TAMs counteract the loss of other pro-tumor TAM states in *Folr2*^*−/−*^ mice and may serve as a compensatory mechanism of immunosuppression.

Our data suggest that folate is a key but limited nutrient in the TME and that FRβ allows TAMs to obtain folate in this environment. Given that FRβ transports folate via endocytosis instead of transporter-mediated conformational changes across the plasma membrane, FRβ is likely not the highest capacity transporter of folate in high folate concentrations. We speculate that only in low folate conditions does the increased affinity of FRβ for folate become important, suggesting that FRβ+ TAMs might have a unique vulnerability to FRβ inhibition because of the low folate concentration within the TME.

## Methods

### Mice

Wild-type C57BL/6J (strain #000664) female mice were initially purchased from The Jackson Laboratory. *Folr2*^−/−^ mice were generated on the C57BL/6J genetic background by the UNC Animal Models Core Facility by micro-injection of CRISPR/Cas9-engineered embryos into pseudo-pregnant dams. In brief, guide RNAs 5g67B (5’-GCACTCCACTCTGTATCAGC-3’) and 3g74B (5’-CGGTTCATCCACCTTCTAGG-3’) designed to introduce double-strand breaks at sites flanking the entire *Folr2* coding sequence (exons 3–6) were cloned into T7-tRNA-gRNA constructs (UNC Animal Models Core). After gRNA validation, C57BL/6J zygotes were microinjected with 400nM high-fidelity eSpCas9 protein (UNC Animal Models Core/UNC Protein Expression and Purification Core Facility), 600nM of each T7-tRNA-gRNA construct and 20ng/μl of donor vector DNA in microinjection buffer (5 mM Tris-HCl pH 7.5, 0.1 mM EDTA). The donor vector DNA contained a 984bp 5’ homology arm, the 4348bp critical region flanked on both sides by loxP sites, and a 1005bp 3’ homology arm. Injected embryos were implanted into recipient pseudopregnant B6D2F1 females. Resulting pups were screened by PCR and confirmed by genetic sequencing for the presence of the floxed allele (i.e. successful incorporation of donor vector DNA at double-strand break sites) as well as the deletion allele (i.e. unsuccessful incorporation of donor vector DNA at double-strand break sites). Pups were bred to generate *Folr2*^*−/−*^ and control animals. Genotyping primers were designed outside of Exon 3 and Exon 6 such that a product was only produced under normal PCR conditions if the deletion allele was present. The following PCR reactions were used:

**Table:** 

Assay	Forward Primer	Reverse Primer	Expected Amplification Band	Polymerase and cycler program
*Folr2* WT	Folr2-SRF1	1159Folr2-3SqR1	WT: 326 bp	Taq: TTD5830
*Folr2* DEL	Folr2-5ScF1	1159Folr2-3SqR1	*Folr2* KO: 285 bp WT: >4.6 kbp	Taq: TTD5830

**Table:** 

Primer Name	Sequence
Folr2-5ScF1	CACTGTCAGGATGGGCTGTCA
Folr2-SRF1	CTACCCTTGGTCTGGTTCAAC
1159Folr2-3SqR1	GAAGCAACTAGATTCCTACAGGCTAC

**Table:** 

TTD5830 (Taq Touchdown)		
1.	95°C	2 min
2.	95°C	30 sec
3.	72°C	30 sec −1°C/cycle
4.	72°C	30 sec
5.		
6.	95°C	30 sec
7.	58°C	30 sec
8.	72°C	30 sec
9.		
10.	72°C	5 min

### Cell culture

B16F10 and MC38 melanoma cell lines were provided by Dr. Arlene Sharpe. B16F10-ova cell lines were made as described.^47^ MCA205 cells were provided by Dr. Jessica Thaxton. B16F10 and B16F10-OVA tumor cell lines were cultured in high glucose Dulbecco’s Modified Eagle Medium (DMEM) supplemented with 10% v/v fetal bovine serum (GeminiBio) and 1% v/v penicillin/streptomycin (Gibco). MC38 tumor cells were cultured in high glucose DMEM supplemented with 10% v/v fetal bovine serum (GeminiBio), 1% v/v penicillin/streptomycin (Gibco), and 1% v/v MEM-NEAA (Gibco). MCA205 tumor cells were cultured RPMI 1640 media (Gibco) supplemented with 10% v/v fetal bovine serum (GeminiBio), 1% v/v penicillin/streptomycin (Gibco), 1% v/v MEM-NEAA (Gibco), and 1mM sodium pyruvate (Gibco). YUMM1.7 cells were cultured DMEM/F-12 media (Gibco) supplemented with 10% v/v fetal bovine serum (GeminiBio), 1% v/v penicillin/streptomycin (Gibco), and 1% v/v MEM-NEAA (Gibco). The LookOut Mycoplasma PCR Detection Kit (Sigma MP0035) was used to periodically verify that all cell lines were negative for mycoplasma contamination. For BMDM cultures, C57BL/6J control or *Folr2*^*−/−*^ mice were euthanized and whole bone marrow flushed from femurs and tibias in cold RPMI 1640 + 2% v/v fetal bovine serum and subsequently filtered through a 70μm cell strainer. RBCs were lysed with ACK lysis buffer (Gibco) and live cells counted. Cells were resuspended in folic acid-free RPMI-1640 media (Gibco) supplemented with 10% v/v dialyzed fetal bovine serum (GeminiBio 100–108), 1% v/v pen-strep (Gibco), 1% v/v MEM-NEAA (Gibco), 1mM sodium pyruvate (Gibco), 10mM HEPES (Corning), and 55μM 2-ME (Gibco) and 20ng/mL M-CSF (Peprotech) and plated in non-TC treated dishes. Folic acid (Sigma-Aldrich) was added to culture media to a final concentration of 10ng/mL (“low folate”) or 1000ng/mL (“high folate”) where indicated. Half media changes and fresh M-CSF additions were performed every 2–3 days for a total of 6–7 days in culture. Terminally differentiated macrophages were confirmed >95% CD11b^+^F4/80^+^ at this time (data not shown). For M(IL-4) BMDMs, 20ng/mL IL-4 was added for the final 24 hours of culture prior to downstream analysis. Mouse T cells were isolated from C57BL/6J spleens by CD8^+^ MACS negative selection (Miltenyi), counted, and cultured in T cell media (TCM) (RPMI-1640 (Gibco) media supplemented with 10% FBS (GeminiBio), 1% penicillin/streptomycin (Gibco), 10mM HEPES (Corning), 55μM 2-mercaptoethanol (Gibco)). TAMs and other myeloid cells were isolated from tumors using CD11b^+^ MACS beads (Miltenyi) and cultured in folate-free RPMI-1640 media (Gibco) supplemented with 10% v/v dialyzed fetal bovine serum (GeminiBio 100–108), 1% v/v pen-strep (Gibco), 1% v/v MEM-NEAA (Gibco), 1mM sodium pyruvate (Gibco), 10mM HEPES (Corning), 55μM 2-ME (Gibco), and 10ng/mL folic acid.

### *In vivo* tumor studies

Tumor cells were cultured to approximately 80% confluency, lifted from plates with 0.25% trypsin + EDTA (Gibco), and live cells counted. Cells were diluted to a concentration of 10^7^ cells/mL in 1x HBSS (Gibco) and 100uL (1×10^6^ cells) were injected subcutaneously into the flanks of male or female 8–12 week old control or *Folr2*^−/−^ mice. Mice were monitored every 2–3 days and tumor growth measured by digital calipers, with tumor volume estimated by the formula (π*(length*width^2^))/6. Mice were fed low folate diet (estimated 0.2mg/kg; Inotiv TD.95247) or high folate (8.6mg/kg folic acid; LabDiet Select Rodent 50 IF/6F Auto) chow diets for at least 4 weeks prior to tumor implantation. Mice were treated with 100μg anti-PD-1 (clone 29F.1A12, BioXCell) or isotype control antibody (clone 2A3, BioXCell) by intraperitoneal injection on the indicated days after tumor implantation. CD8^+^ T cell depletion was accomplished by treating mice with 200μg anti-CD8a (clone 2.43, BioXCell) or the appropriate isotype control antibody (clone LTF-2, BioXCell) starting the day prior to tumor implantation and treating every 2–3 days. For intratumoral BMDM transfer studies, B16F10-OVA tumor cells were cultured and implanted as above into C57BL/6J mice fed a high folate diet. Concurrently, wild-type or *Folr2*^*−/−*^ BMDMs were cultured in 10ng/mL folic acid media for 8 days. When tumors reached approximately 100mm^3^, 1×10^6^ wild-type or *Folr2*^*−/−*^ BMDMs were injected intra-tumorally. Mice were determined to reach a humane endpoint and euthanized if tumor volume exceeded 2000mm^3^, tumor length surpassed 20mm, or body condition score was ≤ 2 or ≥ 4.

### *Ex vivo* human and mouse tumor interstitial fluid and tumor-infiltrating leukocyte analysis

Melanoma patients undergoing metastasis resection to develop a TIL cellular product (Lifileucel) were consented to donate extra tumor tissue for research studies (IRB #90–0573, LCCC 9001 biobanking protocol and IRB #25–0865). Tumors were delivered on ice in PBS to the Miller lab immediately after collection. After blotting dry, the tumors were placed on a 20μm nylon net mesh filter over the opening of a 5mL eppendorf tube and spun (400rcf × 15min at 4°C) to collect tumor interstitial fluid, in which folate was measured by ELISA (see below). Tumors were then mechanically minced and placed in digestion media (RPMI-1640 (Gibco) + 2% FBS (GeminiBio) + 2mg/mL collagenase P (Sigma) + 50mg/mL DNase I (Sigma)) for 30 minutes at 37°C. Fully digested tumors were vortexed, filtered through a 70μm cell-strainer, and washed in cold R2 (RPMI-1640 (Gibco) + 2% v/v FBS (GeminiBio)). Immune cells were isolated using MACS (CD45^+^ TIL or CD11b^+^) bead-based positive selection (Miltenyi) to purify immune cells for flow cytometry analysis. For murine studies, after euthanasia of the mice tumors were dissected and TIF harvested. Tumors were then minced and digested as above, followed by isolation of immune cells by MACS (CD45^+^ or CD11b^+^) bead-based positive selection.

### Isolation of macrophages from mouse organs

To isolate resident tissue macrophages from mouse organs, 8–12 week old C57BL/6J mice that had been placed on folate-deficient chow for 4.5 – 5.5 weeks were euthanized and kidneys, lungs, livers, and spleens removed. For kidneys, lungs and livers, organs were mechanically minced using dissection scissors and digested in 5mL of organ dissociation media (R2 + 1mg/mL collagenase I (Sigma) + 50ug/mL DNase I (Sigma)) for 45min at 37°C, with periodic vortexing at 15min intervals. Kidney, lung and liver suspensions were pulse vortexed, filtered through a 70μm cell strainer, and RBC lysis was performed using ACK lysis buffer (Gibco). Resulting single cell suspensions were CD45+ MACS positively selected (Miltenyi). For spleen processing, organs were mashed through a 70μm cell strainer to generate a single cell suspension and RBC lysis was performed using ACK lysis buffer (Gibco). Lysed spleens were spun down, washed, and refiltered through a 70μm cell strainer.

### *In vitro* and *ex vivo* macrophage functional studies

Wild-type or *Folr2*^*−/−*^ BMDMs were cultured as above in low or high folate media for 5–6 days, followed by 24 hours with 20ng/mL IL-4 (Peprotech). For macrophage cytokine production measurements, 1X Protein Transport Inhibitor Cocktail (eBioscience) containing Brefeldin A and Monensin was added to the wells 15–18 hours prior to harvest to sequester cytokines intracellularly. BMDMs were then lifted from the plates, counted, and extracellular and intracellular flow cytometry performed. For T cell suppression assays, CD8^+^ T cells from C57BL/6J spleens were isolated as above. Purified CD8^+^ T cells were resuspended to a concentration of 5×10^6^ cells/mL in pre-warmed PBS and labeled with Cell Trace Violet (CTV) (ThermoFisher Scientific) at a 1:1000 dilution factor for 20 minutes at 37°C. Cell staining was neutralized with 5X volume of complete TCM and 5×10^4^ stained CD8^+^ T cells were plated on non-TC treated U-bottom 96-well plates that had been pre-coated for 16–24 hours at 4°C with 1mg/mL agonistic anti-CD3 (eBioscience) and anti-CD28 (eBioscience) antibodies diluted in PBS. Different numbers of BMDMs were added into the wells for the indicated BMDM:T cell ratios. For the final 6 hours prior to T cell coculture harvest, 1X Protein Transport Inhibitor Cocktail was added to the wells. After a total of 72 hours, all cells were collected and analyzed by flow cytometry to assess CTV dilution, T cell surface phenotype, and intracellular cytokine production. Where indicated, BMDMs were pre-treated for 4–6 days with 10μM VBIT-4 (Selleck Chemicals), 5mM N-acetyl-L-cysteine (Sigma-Aldrich), 5mM L-glutathione (Sigma-Aldrich), or 20μM MitoTEMPO (Sigma-Aldrich) prior to co-culture with T cells or cytokine measurements. Finally, myeloid cells were isolated from B16F10 tumors as above and cultured for 18 hours in folic acid-free RPMI-1640 media (Gibco) supplemented with 10% v/v dialyzed fetal bovine serum (GeminiBio 100–108), 1% v/v pen-strep (Gibco), 1% v/v MEM-NEAA (Gibco), 1mM sodium pyruvate (Gibco), 10mM HEPES (Corning), 55μM 2-ME (Gibco), and 10ng/mL folic acid (Sigma-Aldrich). For the final 6 hours of culture 1X Protein Transport Inhibitor Cocktail was added to the media, then cells lifted from the plates and analyzed by flow cytometry.

### *In vitro* BMDM survival and proliferation studies

To measure BMDM survival *in vitro*, we cultured wild-type and *Folr2*^*−/−*^ BMDMs in media containing different concentrations of folic acid for 6–8 days. Cells were lifted, stained with flow antibodies, washed, and then stained with Annexin-V-FITC (BioLegend 640906) diluted 1:200 in Annexin-V Binding Buffer (BioLegend 422201) for 15min at 4C. Prior to flow cytometry, cells were incubated with 7-AAD per the manufacturer’s instructions (BioLegend 420404). To measure proliferation *in vitro*, we added 1mM BrdU to wild-type or *Folr2*^*−/−*^ BMDMs cultures 9 hours prior to harvest. Cells were lifted on day 2, 4, or 6 after bone marrow collection. Cells were fixed and permeabilized using the BD Pharmingen FITC BrdU Flow Kit (BD Pharmingen 559619), then stained with anti-BrdU-FITC antibodies for flow cytometry analysis.

### Flow Cytometry

BMDMs and immune cells isolated from mouse tumors or organs for flow cytometry were blocked in MACS buffer (PBS + 2% v/v fetal bovine serum + 0.4% v/v EDTA) + TruStain FcX anti-mouse CD16/32 (BioLegend) and 2% normal mouse serum (Jackson ImmunoResearch) for at least 20 minutes at 4°C. Human immune cells were blocked in MACS buffer + TruStain FcX anti-human CD16/32 (BioLegend). Samples were then stained for surface proteins in MACS buffer for 30 minutes at 4°C. Live/Dead fixable viability dyes (ThermoFisher Scientific) were included for dead cell exclusion. Staining master mixes were diluted in MACS buffer or BD Horizon Brilliant Stain Buffer (BD) for panels that included multiple brilliant-violet conjugated antibodies. Intracellular staining was then performed as needed by fixing and permeabilizing the cells using the eBioscience IC Fixation & Permeabilization Buffer Set (ThermoFisher Scientific) according to the manufacturer instructions. Permeabilized cells were again blocked with MACS buffer + blocking reagents listed above and then stained with antibodies diluted in 1X eBioscience Permeabilization Buffer (ThermoFisher Scientific). For analysis of mitochondrial function and redox dyes, cells were stained with 100nM tetramethylrhodamine (TMRM) (ThermoFisher Scientific), 100nM MitoTracker Green (ThermoFisher Scientific), 3μM MitoSox Red (ThermoFisher Scientific), or 5μM CellRox (ThermoFisher Scientific), each diluted in PBS, for 30 minutes at 37°C followed by subsequent washing and blocking/cell surface antibody staining. Flow data was collected on LSRFortessa (BD Biosciences), FACSymphony A3 (BD Biosciences), or Aurora (Cytek Biosciences) flow cytometers after addition of CountBright Absolute Counting Beads (ThermoFisher Scientific) to quantify cell numbers. Data was analyzed on FlowJo (v10.10.0, TreeStar), with compensation performed manually using single-color control samples. Gating was assigned using fluorescence-minus one and/or isotype-matched control antibody stained samples. For mouse samples, the following gating strategies were used: kidney, liver lung and splenic macrophages were gated on Live CD45^+^CD64^+^F4/80^+^ cells. TAMs were gated on Live CD45^+^CD11b^+^Ly6G^−^Ly6C^−^F4/80^+^. Monocytes were gated on Live CD45^+^CD11b^+^Ly6G^−^Ly6C^+^F4/80^−^ cells. Neutrophils were gated on Live CD45^+^CD11b^+^Ly6G^+^Ly6C^+^ cells. T cells were gated on Live CD45^+^CD11b^−^TCR-β^+^ cells. NK cells were gated on Live CD45^+^CD11b^−^TCR-β^−^NK1.1^+^ cells. For human samples, TAMs were gated on Live CD45^+^CD64^+^CD68^+^.

### ASC puncta immunofluorescence and quantification

Wild-type and *Folr2*^*−/−*^ M(IL-4) BMDMs were plated on sterile glass coverslips (72230–01, Electron Microscopy Sciences) for 24 hours in 24 well plates. Cells were maintained in low or high folate media until washing and fixation in fresh 4% PFA-PBS (15710, Electron Microscopy Sciences) for 10 minutes. Cells were permeabilized with 0.1% Saponin (S4521–25G, Sigma) maintained in all subsequent buffers. Cells were blocked with 5% normal goat serum (005-000-121, Jackson ImmunoResearch) and autofluorescence quenched with 50mM NH_4_Cl. Cells were stained with anti-ASC (AG-25B-0006-C100, Adipogen, 1:100), AlexaFluor 488 anti-rabbit secondary (A32731, Invitrogen, 1:400), DAPI 405 (D3571, ThermoFisher, 1:1000), and AlexaFluor 647-conjugated Phalloidin (8940S, Cell Signaling Technology, 1:400). Specks were imaged by confocal microscopy (ZEISS LSM900). Briefly, ASC specks were quantified by randomly generating at least five tiles in ZEN blue software. ASC puncta and nuclei were counted and summed across all tiles for a given replicate. Finally, ratios of total ASC puncta to nuclei were calculated.

### Mitochondrial immunofluorescence

Wild-type and *Folr2*^*−/−*^ M(IL-4) BMDMs were cultured in low folate media until washing and fixation. Cells were fixed in paraformaldehyde (4%) in PBS for 15 minutes and then permeabilized with TritonX-100 (0.1%) for 15 minutes. BMDMs were blocked in 2% BSA in PBS for 1 hour before staining with Phalloidin AlexaFluor 405 (A30104, Invitrogen) mouse anti-TOM20 (sc-17764, Santa Cruz), or anti-LAMP1 (9091, Cell Signaling) antibodies in 1% BSA in PBS for 1 hour at room temperature. Cells were washed with 1x PBS and incubated with the secondary antibodies goat anti-mouse IgG (H+L) AlexaFluor 488 (A32723TR, Invitrogen) and goat anti-rabbit IgG (H+L) AlexaFluor 594 (A32740, Invitrogen) in 1% BSA in PBS for 1 hour at room temperature. Cells were imaged using the Zeiss LSM-880 confocal microscope with ZEN acquisition software (Zeiss). Image analysis was performed in Fiji ImageJ.

### Enzyme-linked immunosorbent assays

For cytokine secretion, media was harvested from wild-type or *Folr2*^*−/−*^ M(IL-4) BMDM cultures, centrifuged at 400 rcf for 5 minutes to remove cellular contaminants, and stored at −80°C for subsequent thawing and analysis. Secreted IL-1β was quantified from generated supernatants using the Mouse IL-1 beta/IL1F2 DuoSet ELISA kit (R&D Systems) following manufacturer instructions. For intracellular folate concentration, adherent wild-type or *Folr2*^*−/−*^ BMDMs were harvested, washed, and lysed on a nutator for 30 minutes at 4°C in Cell Lysis Buffer II (ThermoFisher Scientific) supplemented with 50mM ascorbic acid (Sigma-Aldrich) and Pierce Protease Inhibitor (ThermoFisher Scientific), with pulse vortexing at 10 minutes intervals. After cell lysis, a small aliquot of each sample was extracted for protein quantification using BradFord Protein Assay (BioRad). The remainder of each lysate was heated at 100°C for 5 minutes and subsequently centrifuged at 14000 rcf for 10 minutes at 4°C. The resulting supernatant containing extracted folate species was transferred to a chilled eppendorf tube and used to analyze intracellular folate levels using the Folic Acid ELISA Kit (CellBio Labs). To control for cell differences between samples, quantified intracellular folate levels were normalized by total protein concentrations on a per sample basis.

### Transmission electron microscopy

Macrophage samples were collected and fixed in 2.5% glutaraldehyde/2% paraformaldehyde in 0.15 M sodium phosphate buffer, pH 7.4 for 1 hour at room temperature. Cells were then pelleted and washed with 0.15 M sodium phosphate buffer pH 7.4 three times for 10 minutes each then incubated in 1% osmium tetroxide in 0.15 M sodium phosphate buffer for 1 hour at room temperature. Cell pellets were washed three times with deionized water then dehydrated through an increasing ethanol series (30%, 50%, 75%, 90%, 100%, 100%, 100%, 10 minutes each) followed by two rounds of propylene oxide for 15 minutes each. Pellets were infiltrated with a 1:1 mixture of propylene oxide:Polybed 812 epoxy resin for several hours (Polysciences, Inc., Warrington, PA) followed with incubation in 100% Polybed 812 epoxy resin overnight. Resin was exchanged once more, then allowed to cure at 60°C. Ultrathin sections (80 nm) were cut using a diamond knife on a Leica UCT7 ultramicrotome and applied to 200 mesh copper grids. Grids were stained with 4% aqueous uranyl acetate for 12 minutes followed by Reynold’s lead citrate for 8 minutes (Reynolds, 1963). Samples were viewed using a JEOL 1400 Flash transmission electron microscope operating at 80 kV (JEOL USA, Inc.) and images were acquired with an AMT NanoSprint61 ActiveVu: High Resolution CMOS TEM camera (Advanced Microscopy Techniques).

### Seahorse XF Mito Stress Test

Wild-type and *Folr2*^*−/−*^ BMDMs were cultured in low folate media for 6 days, followed by 24 hours with IL-4, as above. Equal numbers of wild-type and *Folr2*^*−/−*^ BMDMs (1×10^5^ cells/well) were plated onto Agilent plates coated with Cell-Tak (Corning) for at least 30 minutes prior to analysis. The Agilent Seahorse XF Cell Mito Stress Test was performed as per manufacturer’s instructions, with 12 samples per condition. The following final concentrations of mitochondrial toxins were used: 1.5μM oligomycin, 1.5μM FCCP, 2μM Rotenone + 2μM Antimycin A. Individual wells were excluded from analysis if they exhibited an outlying OCR curve, suggesting port injection failure.

### Immunoblotting

Cell lysates were prepared with RIPA Lysis and Extraction Buffer (#89900, Thermo Fisher) containing protease inhibitor cocktail (#A32953, Thermo Fisher) and phosphatase inhibitors cocktail (#4906845001, Roche). Cell lysates were kept on a rotator at 4°C for 30 minutes, and centrifuged at 10,000 rpm for 10 minutes at 4°C. Protein concentrations were measured using a BCA assay kit (#23227, Thermo Fisher) following the manufacturer’s protocol. Equal amounts of total protein were separated on 4–12% or 15% Bolt Bis-Tris Plus gels (Invitrogen) and transferred 250mA for 90 minutes at room temperature (RT) onto 0.45 μm PVDF membranes (Millipore). Membranes were blocked with 5% (w/v) non-fat milk in Tris-buffered saline containing 0.05% Tween 20 (TBST) for 1 hour at RT, then incubated overnight at 4°C with primary antibodies used at 1:1000 concentration unless otherwise indicated: anti-STING (Cell Signaling, #13647), anti-phospho-STING Ser365 (Cell Signaling, #72971), anti-TBK1 (Cell Signaling, #3504), anti-phospho-TBK1 Ser172 (Cell Signaling, #5483), anti-phospho-NFkB p65 Ser536 (Cell Signaling, #3033), anti-NFkB p65 (Cell Signaling, #8242), anti-LC3B (Cell Signaling, #2775) and anti-Vinculin (1:10,000; Sigma-Aldrich, #V9131). After three 5-minute washes in TBST, membranes were incubated for 1 hour with the corresponding HRP-conjugated secondary antibodies (1:2000 anti-rabbit, Cell Signaling, #7074P2; 1:5000 anti-Mouse, GE Healthcare, Amersham Life Sciences #NA931; 1:5000 anti-Rabbit, GE Healthcare, Amersham Life Sciences #NA934). Following three 5-minute washes, protein bands were visualized and quantified by chemiluminescence using the ChemiDoc MP Imaging System (#12003154, Biorad).

### Mouse TAM and BMDM bulk RNA-seq

Wild-type and *Folr2*^*−/−*^ M(IL-4) BMDMs were lifted and washed for RNA isolation, with separate tissue culture plates of cells used for 3 biologic replicates. For mouse TAMs, B16F10-OVA tumors were implanted into wild-type or *Folr2*^*−/−*^ mice fed low or high folate diet. To ensure adequate cell number per sample, CD11b^+^ MACS-selected cells were randomly pooled from 3–7 individual tumors within folate diet and mouse genotype to generate a total of 3 independent biologic replicates per group. After 16 days of growth, tumors were extracted and myeloid cells isolated by CD11b^+^ MACS positive selection beads (Miltenyi), followed by flow sorting for TAMs gated as Live CD45^+^CD11b^+^Ly6G^−^Ly6C^−^F4/80^+^ cells. Total RNA was isolated from cell pellets using a Zymo RNA Extraction Kit following the manufacturer’s protocol. Total RNA quality was analyzed using an RNA ScreenTape (Agilent 5067–5576) and TapeStation 4200 (Agilent G2991AA) and the quantity was measured using an RNA Broad Range kit (Invitrogen Q10211) and Qubit Flex Fluorometer (Thermo Scientific Q33327). mRNA sequencing libraries were prepared from up to 1000ng of total RNA using a TruSeq Stranded mRNA Library Prep Kit (Illumina 20020595) following the manufacturer’s protocol (Illumina 1000000040498). mRNA library quality and quantity were measured using a D1000 DNA Screentape (Agilent 5067–5582) and a Qubit 1X dsDNA High Sensitivity Assay Kit (Thermo Fisher Q33231), combined at equal molar ratios, and denatured following the manufacturer’s protocol (Illumina 200047032). The pool was sequenced on an Illumina NextSeq2000 P3 flow cell with a 2×50bp paired-end configuration to a designed depth of 35 million clusters per library on average and cDNA generated using Illumina barcodes.

Bulk RNA-seq data were processed using Nextflow v22.04.4 (build 5706). Sequencing reads were aligned to the mouse reference genome using STAR, with the GRCm38.p3 genome assembly and the corresponding GENCODE M9/Ensembl release 84 gene annotation. Transcript-level abundance was quantified using Salmon against the corresponding reference transcriptome. The resulting expression estimates were used for downstream gene expression and differential expression analyses. For differential expression analysis, genes were retained if they were expressed at a minimum of 25 counts in all mice within at least one experimental group. Differential gene expression analysis was performed using the DESeq2 package. The resulting lists of differentially expressed genes were subsequently analyzed for gene set enrichment using Gene Set Enrichment Analysis (GSEA), including gene sets from the Hallmark, C2, and C5 collections.

### Mouse single-cell RNA-seq

B16F10 tumors were implanted into wild-type or *Folr2*^*−/−*^ mice fed a low folate diet. On day 17 of tumor growth, tumors were extracted and all tumor-infiltrating immune cells isolated by CD45^+^ TIL MACS positive selection beads (Miltenyi) as above, followed by flow sorting for total live CD45^+^ cells. 5 mice of each genotype were treated as independent replicates and labeled with individual TotalSeq-B Anti-Mouse Hashtag Antibodies (Biolegend) then sorted separately prior to pooling of an estimated 8000 cells from each of the 10 samples (5 wild-type, 5 *Folr2*^*−/−*^). Pooled samples were loaded across 2 GEM-X 3’ chip wells using the Chromium Controller (10x Genomics). Feature Barcoding and Gene Expression libraries were generated per Chromium GEM-X Single Cell 3’ v4 protocol and sequenced at a 1:9 ratio (respectively) on one lane of an Illumina NovaSeq X 25B flow cell (r1: 28bp, i7: 10bp, i5: 10bp, R2: 90bp).

Data were mapped and demultiplexed using the Cell Ranger Multi Unified Runner (10x Genomics) against the GRCm39–2024-A mouse reference genome. Cells identified as singlets were subsequently used for downstream analysis. Data preprocessing, normalization, integration, and clustering were performed using the Seurat package (v. 5.4.0) in R (v. 4.4.1). We excluded cells that did not meet any of the following criteria: fewer than 1,000 counts, fewer than 300 genes, a count-to-gene ratio above 7.5, a mitochondrial count percentage above 5%, or a ribosomal count percentage above 30%. We further applied DoubletFinder and filtered out putative doublets. All samples were then merged into a single Seurat object. The resulting expression matrix contained 29,449 cells, including 15,032 KO and 14,417 WT cells.

Dimensionality reduction and clustering were performed using Seurat’s standard clustering workflow, utilizing the functions “FindVariableFeatures,” “RunPCA,” and “RunUMAP.” Where applicable, the first 30 principal components were used. Unsupervised clustering was performed using the “FindNeighbors” and “FindClusters” functions. We first identified major cell types and found that a resolution of 0.01 was sufficient to distinguish between these clusters. Cells identified as myeloid cells, lymphocytes, and neutrophils were then analyzed as individual subsets. For each subset, we tested several clustering resolutions and selected the resolution based on the correspondence of clusters with known biological markers. Differential expression analyses between clusters and to assess the effect of the knockout were performed using a Wilcoxon rank-sum test implemented through the “FindMarkers” function. The resulting lists of differentially expressed genes were further analyzed for signature enrichment using GSEA, including signatures from the Hallmark, C2, and C5 collections. Cell-level scores for several of the more closely studied signatures were calculated using the UCell package with kNN smoothing. Trajectory analysis was performed using Monocle3 on the neutrophil subset.^54^

### Human rapid warm autopsy specimens

Warm autopsies were performed as previously described.^46^ In brief, an Autopsy Authorization Form was signed by the patient or healthcare power of attorney prior to death of the patient. Rapid autopsy was performed at the University of North Carolina at Chapel Hill and tissue samples collected no longer than 8 hours from death. Samples were collected for metabolomics by flash freezing tissues and storing them at −80°C. Formalin Fixed, paraffin embedded (FFPE) samples were collected for Visium v1 spatial analysis (10x Genomics).

### Metabolomics sample preparation

Cells were prepared for metabolomics analysis according to previously described methods.^55,56^ In brief, wild-type and *Folr2*^*−/−*^ BMDMs were cultured in low or high folate media for 5 days, followed by 24 hours with IL-4, as above. On day 6 cells were washed 2x with ice-cold PBS, then quenched and lysed in −20°C acetonitrile and ice-cold H2O. Separate plates of BMDMs were used for 6 biologic replicates. Samples were vortexed, centrifuged at 16,000g for 10 min at 4°C, and supernatants were dried by speed vac and reconstituted in a volume of 95:5 water:methanol that was proportional to protein concentration. Tissue samples were placed in tubes with ceramic beads and homogenized in a volume of isopropanol proportional to tissue weight (2 μL/mg of tissue). Supernatants were dried by speed vac and reconstituted in 100 uL of 95:5 water:methanol. Quality control study pools (QCSPs) were created by combining aliquots of each study sample. Additionally, method blanks were created by aliquoting LC-MS grade water and performing the same extraction steps as the study samples. Final extracts were analyzed by ultra high performance liquid chromatography-high resolution mass spectrometry (UHPLC-HRMS) at the Metabolomics and Exposome Laboratory at the University of North Carolina at Chapel Hill’s Nutrition Research Institute.

### UHPLC-HRMS metabolomics data acquisition

Untargeted UHPLC-HRMS metabolomic profiling was conducted on a Vanquish UHPLC system coupled to a Q Exactive HF-X Mass Spectrometer (ThermoFisher Scientific), using previously described methods.^55,56^ Samples were placed in the autosampler in a randomized sequence, with quality control samples (QCSP) and blank injections interspersed throughout the study samples, and 5 μL was injected from each sample. Metabolite separation was performed using an HSS T3 C18 column (2.1 × 100 mm, 1.7 μm, Waters Corporation) maintained at 50°C. The mobile phase consisted of water with 0.1% formic acid (v/v) as solvent A and methanol with 0.1% formic acid (v/v) as solvent B. A gradient elution was applied, beginning at 1% B and rising to 100% B over 16 minutes, followed by a 4-minute hold, all at a constant flow rate of 400 μL/min. Full-scan mass spectra were collected across a range of 70–1050 m/z in both positive and negative mode, while MS/MS spectra were obtained through data-dependent acquisition, selecting the 20 most abundant ions for fragmentation. Raw data files were processed using Progenesis QI (version 2.1, Waters Corporation, Milford, MA, USA), and peaks showing greater average intensity in blank samples than in QC samples were excluded to eliminate background noise.

### Compound identification and annotation

Metabolite annotation was performed by matching all peaks to an in-house library of reference standards and public metabolite databases, including the NIST Tandem Mass Spectral Library and the Human Metabolome Database (HMDB). A peak was considered a match when it satisfied one or more criteria: accurate mass within 5 mg/kg (MS), fragmentation spectra with a similarity score greater than 30% (MS/MS), or retention time falling within ±0.5 minutes of the reference value for in-house matches only. To convey the evidence basis for each match, we applied an ontology system described previously.^55,56^ In-house library matches were labeled as OL1 when matched by MS, MS/MS, and RT together; OL2a when matched by MS and RT; and OL2b when matched by MS and MS/MS. Matches from public databases were labeled PDa (MS and MS/MS), PDc (MS and isotope pattern similarity), or PDd (MS alone). Notably, this workflow cannot always resolve certain isomeric structures from one another.

### Metabolomics analysis

Data was normalized between samples based on pooled standards. Basic analyses were conducted using sPLS-DA to assess the separation of experimental groups, as well as pooled samples, based on all positively and negatively charged metabolite measurements following basic filtering to remove metabolites in the bottom 10% of the IQR and the bottom 5% of expression. Our imputation strategy was based on the assumption that values below the detection threshold across the entire experimental group should be imputed as 10 - a very low value, but not too small to create an artifact during further data transformation. When most measurements within a group were missing, missing values were imputed as one-fifth of the mean value calculated from the non-missing samples. The remaining missing values were imputed using the Bayesian Principal Component Analysis (BPCA) approach. The data were subsequently normalized on a per-sample basis using median normalization, followed by log10 transformation and Pareto scaling. To assess differences between experimental groups, we first filtered the data to identify the most robust metabolite measurements, prioritizing those that matched one of the following ontology levels in the specified order: OL1, OL2a, OL2b, PDA, PDB, PDC, and PDD. Data preparation and analysis were performed using R version 4.4.1 and MetaboAnalyst version 6.0. For statistical analysis, we performed t-tests and estimated log fold changes (logFC). Finally, pathway analysis was performed to identify pathways associated with the observed differences between experimental groups.

### Human single-cell analysis

Human melanoma single-cell RNA-seq datasets were downloaded, split based on sample IDs, normalized using SCTransformm and samples integrated using the RPCA method (https://doi.org/10.48804/GSAXBN, GSE120575).^41,42^ The two datasets were then integrated using the RPCA method. The integrated object was scaled and clusters identified using the Louvain algorithm. Myeloid cells were identified using canonical genes and reclustered to identify myeloid subsets.

### Human spatial transcriptomic analysis

Regions of interest from FFPE-fixed metastatic melanoma tumor resections were reviewed by a melanoma pathologist based on hematoxylin and eosin (H&E) staining, with a focus on the tumor-stroma interface. Tumors with an adequate amount of melanoma (covering at least 40% of the Visium capture area), devoid of necrosis and pigment, were QCed for RNA quality (DV200 > 30%). 5μm FFPE sample sections are mounted on the Visium FFPE v1 slides, dried overnight and processed for HE staining at (UCLA Translational Pathology Core Laboratory (TPCL)) and library construction (UCLA Technology Center for Genomics & Bioinformatics (TCGB)) according to the manufacturer’s protocol. Single-cell barcoded sequencing libraries were run on an Illumina NovaSeq 6000. Raw FASTQ files along with HE scan images were processed using the Space Ranger pipeline v1.3.0 and GRCh38–2020-A reference transcriptome. The gene counts for each Visium well in each sample were used in the following downstream analyses. The transcriptomic clustering was generated by SpaceRanger using its default graph-based Louvain clustering algorithm. We calculated cell abundances within each Visium well using cell2location,^57^ based on our combined melanoma single-cell RNA-seq datasets.^41,42^

### Human survival analysis

Gene expression data and clinical metadata for Melanoma (SKCM) from The Cancer Genome Atlas (TCGA) were downloaded from the cBioPortal website. Survival analysis was performed using the Kaplan–Meier method, with survival curves estimated using the survfit function. The ratio of *FOLR2*/*CD8A* expressions was binarized into low- and high-expression groups and used as a predictor of survival. To assess the robustness of the findings to the choice of expression threshold, multiple percentile-based cutoffs were evaluated, ranging from the 20th to the 80th percentile (0.2–0.8), with samples categorized into low- and high-expression groups at each cutoff. In addition, Cox proportional hazards regression was performed using continuous, log-transformed *FOLR2* and *CD8A* expression levels as predictors of survival. Sex, age, and body weight were included as additional covariates in the multivariable Cox regression model.

### Statistical analysis

For all tumor growth experiments, at least 5 mice per group were used per experiment with at least 2–3 independent experiments performed. Experimental results were analyzed via unpaired two-sided Student’s t-tests using GraphPad Prism 10. Outlier data points in mouse experiments were identified and removed using Rout outlier tests executed with default Prism parameters. Genomics and metabolomics data were analyzed as discussed above. Statistical significance was signified with asterisks that correspond to the following p values: ns not significant (p > 0.05), * p ≤ 0.05, ** p ≤ 0.01, *** p ≤ 0.001, **** p ≤ 0.0001.

## Supplementary Material

**Supplementary Figure 1. (A)** Flow cytometry gating strategy for human patient melanoma samples. **(B)** tSNE analysis of Live CD45^+^ immune cells from one representative patient sample. **(C)** Flow cytometry gating strategy for mouse tumor samples. **(D)** Flow cytometry gating strategy for mouse organ samples. **(E,F)** *FOLR2* transcript **(E)** and FRβ protein **(F)** expression data from the Human Protein Atlas v25.1 (proteinatlas.org). **(G)** tSNE analysis of Live CD45+ immune cells from 3 pooled B16F10 tumors. Created in BioRender. Zimmerman, M. (2027) https://BioRender.com/lv1kxak

**Supplementary Figure 2. (A-B)** Wild-type and *Folr2*^*−/−*^ mouse and spleen weights from 1 (mouse) or 2 (spleen) independent experiments. Mice were aged 7–12 weeks old. **(C)** Per sample single-cell quality metric scores. **(D)** Heatmaps demonstrating hashtag counts per sample including potential multiplets (diagonal = singlets, off diagonal = multiplets). **(E)** Heatmap of top differentially expressed genes from each major UMAP cluster. **(F)** UMAP clustering of all 29,499 immune cells isolated from wild-type and *Folr2*^*−/−*^ tumors. **(G)** Expression of *Folr2* in individual tumor-infiltrating leukocytes from B16F10 tumors (left) and violin plot of *Folr2* expression by major cell cluster (right). **(H)** Dot plot of key representative genes for each cluster of myeloid cells from Fig. 2E. **(I)** Expression of *Folr2* in each myeloid cell cluster from Fig. 2E. **(J)** GSEA of inflammatory gene signatures in the ranked list of genes differentially expressed between TAMs sorted from wild-type and *Folr2*^*−/−*^ mice. **(K)** scRNA-seq profiles of tumor-infiltrating neutrophils from B16F10 tumors implanted into wild-type or *Folr2*^*−/−*^ mice. UMAP of neutrophil clusters shown. **(L)** Cell density via galaxy plot in UMAP space (left) and quantification of the percentage of cells in each cluster between wild-type and *Folr2*^*−/−*^ mice (right). **(M)** Expression of key genes in individual tumor-associated neutrophils from B16F10 tumors. **(N)** Pseudotime trajectory analysis of neutrophil states using Monocle3.

**Supplementary Figure 3. (A)** Representative flow plot demonstrating intratumoral CD8^+^ T cell depletion with anti-CD8α depleting compared with isotype control antibody treatment. **(B)** Dot plot of key representative genes for each cluster of lymphocytes from Fig. 3F. **(C)** Weighted interactions between clusters. Higher scores (red) indicate higher potential interactions between clusters, lower scores (blue) indicate less interaction between clusters. Separate heatmaps for wild-type and *Folr2*^*−/−*^ mice shown. The per cell difference between these heatmaps is plotted in Fig. 3I.

**Supplementary Figure 4. (A)** Schema of folate transporters expressed in mice. **(B)** Folate transporter gene expression in wild-type or *Folr2*^*−/−*^ mouse TAMs from B16F10-OVA tumors. **(C)** Cell counts of wild-type or *Folr2*^*−/−*^ BMDMs grown for 6 days in folate-free media supplemented with the indicated concentrations of folic acid. **(D)** Flow cytometry quantification of 7-AAD^+^Annexin-V^+^ dead/dying BMDMs from experiment in **(C)**. **(E)** BrdU uptake in wild-type or *Folr2*^*−/−*^ BMDMs grown in 10ng/mL or 1000ng/mL folic acid. BrdU added to media for 9 hours prior to harvest at days 2, 4, or 6 after isolation of bone marrow from mice. **(F)** Plot of signatures significantly enriched (FDR < 0.1) in the comparison of wild-type vs *Folr2*^*−/−*^ TAMs and wild-type vs *Folr2*^*−/−*^ M(IL-4) BMDMs. Normalized enrichment score of each signature plotted on each axis. **(G)** Representative flow plots of PD-1^+^CD44^+^, IFNү, or TNFα expression by CD8^+^ T cells +/− wild-type or *Folr2*^*−/−*^ BMDMs after *in vitro* co-culture. **(H)** Serum folate levels of mice fed a low folate or high folate diet for 4 weeks as measured by folate ELISA. **(I)** Hemoglobin, mean corpuscular volume, and white blood cell counts from blood of mice in **(H)**. **(J)** Tumor growth in wild-type mice fed low folate or high folate chow for four weeks prior to tumor implantation. Pooled data from 2 independent experiments shown. * p < 0.05; ** p < 0.01; *** p < 0.001; **** p < 0.0001; ns not significant. Created in BioRender. Zimmerman, M. (2027) https://BioRender.com/vbzp9l2

**Supplementary Figure 5. (A)** Schema of generation of BMDMs for untargeted metabolomics. **(B,C)** PCA of RNA-seq **(B)** and PLS-DA **(C)** of untargeted metabolomics data from wild-type and *Folr2*^*−/−*^ M(IL-4) or M(M-CSF) BMDMs grow in low folate or high folate media. **(D)** Heatmap of differentially expressed metabolites (FDR<0.1) between wild-type and *Folr2*^*−/−*^ M(IL-4) BMDMs. **(E)** Volcano plot of metabolites comparing wild-type and *Folr2*^*−/−*^ M(IL-4) BMDMs grown in high folate media. **(F)** GSEA of oxidative stress gene signatures in the ranked list of genes differentially expressed between wild-type and *Folr2*^*−/−*^ M(IL-4) BMDMs grown in low folate media. **(G)** Quantification of MitoSox+ wild-type or *Folr2*^*−/−*^ M(IL-4) BMDMs cultured +/− 5mM glutathione (GSH) or 5mM N-acetylcysteine (NAC). **(H)** ASC puncta formation in wild-type and *Folr2*^*−/−*^ M(IL-4) BMDMs grown in low or high folate media detected by immunofluorescence. Blue = DAPI (nuclei), Green = ASC, Red = phalloidin (actin). Representative images (left) and quantification (right). Wild-type BMDMs cultures in 100ng/mL LPS + 20ng/mL IFNу shown as a positive control for ASC puncta formation. White arrows identify individual puncta. **(I)** IL-1β secretion measured by ELISA from wild-type and *Folr2*^*−/−*^ M(IL-4) BMDMs grown in high and low folate media, with M(LPS+IFNу) as a positive control for inflammasome activation. Results shown are averages from triplicate wells across 2 independent experiments. * p < 0.05; ** p < 0.01; *** p < 0.001; **** p < 0.0001; ns not significant. Created in BioRender. Zimmerman, M. (2027) https://BioRender.com/g7hgnxa

**Supplementary Figure 6. (A)** GSEA of mitochondrial gene signatures in the ranked list of genes differentially expressed between wild-type and *Folr2*^*−/−*^ M(IL-4) BMDMs grown in low folate media. **(B)** Quantification of maximal oxygen consumption rate (OCR) and spare respiratory capacity (SRC) from Seahorse experiment shown in Fig. 6C. **(C)** Representative electron microscopy images of wild-type and *Folr2*^*−/−*^ M(IL-4) BMDMs grown in low folate media at lower resolution. Same cells as shown in Fig. 6D. **(D)** Immunoblot of lysates from wild-type and *Folr2*^*−/−*^ M(IL-4) BMDMs grown in low folate media +/− Bafilomycin A1 treatment stained against LC3 and Vinculin.

## Figures and Tables

**Figure 1:**
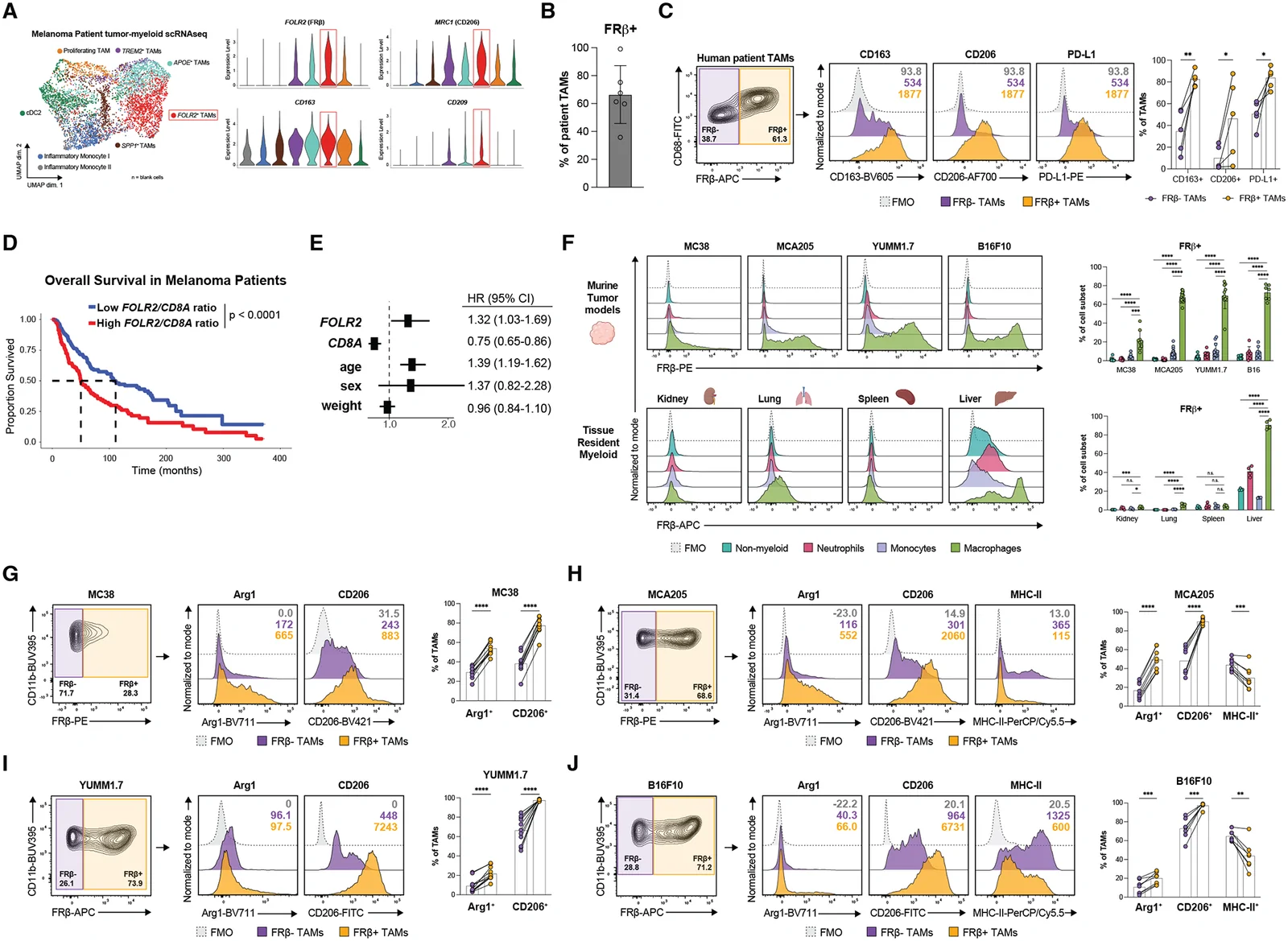
FRβ is expressed by TAMs with an immunosuppressive phenotype and is associated with poor melanoma patient outcomes. **(A)** Single-cell data combined from Sade-Feldman *et al*. and Pozniak *et al*. and subclustered on myeloid cells. *FOLR2*^+^ TAM cluster shown in red; co-expression of *FOLR2* and markers of immunosuppressive macrophages (*MRC1*, *CD163*, *CD209*) shown. **(B)** Percentage of human TAMs expressing FRβ from 6 melanoma tumors isolated from 4 patients undergoing metastatic melanoma resection for Lifileucel treatment. **(C)** Flow cytometry analysis of FRβ, CD163, CD206, and PD-L1 from human patient TAMs. Representative flow plots (left) and summary bar plots (right) from 5 patient tumors (excluding tumor with 100% FRβ+ TAMs). **(D)** Kaplan-Meier estimate of survival in the melanoma (SKCM) cohort of TCGA, stratified as *FOLR2/CD8A* ratio high or low using 60/40 percentile as cutoff value. At all cutoffs between 0.2 and 0.8 we obtained a significant p-value. P value from log-rank test. **(E)** Multivariate Cox analysis of the same dataset accounting for *CD8A*, sex, age, and weight. **(F)** Flow cytometry analysis of FRβ expression by tumor-infiltrating and tissue resident leukocytes across 4 tumor models and 4 organs. Representative histograms (left) and summary bar plots (right) from two independent experiments shown. **(G-J)** Flow analysis of FRβ+ and FRβ− TAMs from MC38 **(G)**, MCA205 **(H)**, YUMM1.7 **(I)** and B16F10 **(J)** tumor models showing expression of Arg1, CD206, and MHC-II in each populations. Representative histograms (left) and summary bar plots from 2 pooled experiments shown (1 experiment for MCA205). * p < 0.05; ** p <0.01; *** p < 0.001; **** p < 0.0001. Created in BioRender. Zimmerman, M. (2027) https://BioRender.com/wk9olvt

**Figure 2.**
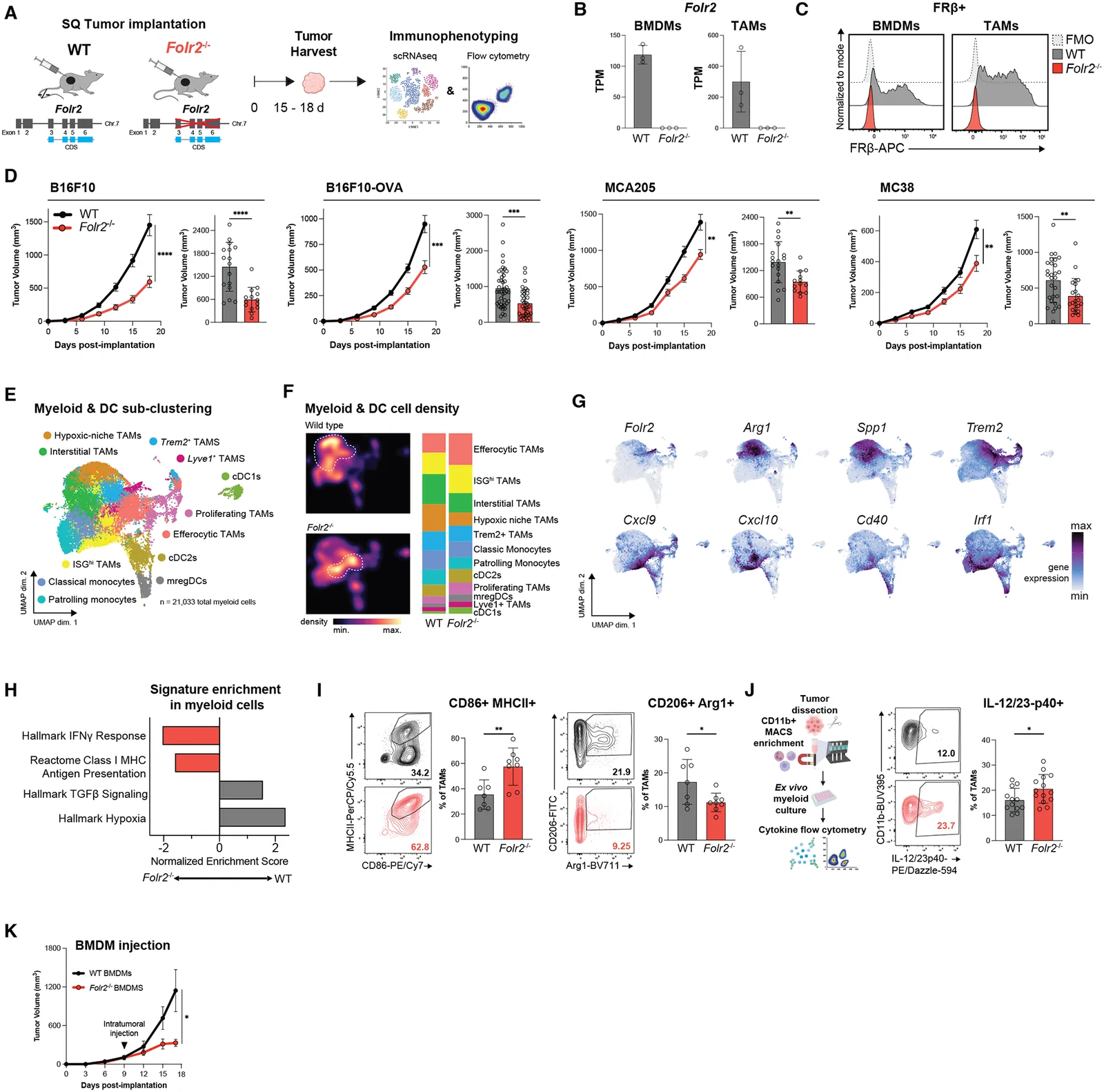
FRβ supports TAM immunosuppressive polarization and function. **(A)** Schema of *Folr2*^*−/−*^ mouse generation and immunophenotyping. **(B)** RNA-seq analysis of *Folr2* expression in bone-marrow derived macrophages (BMDMs) or TAMs sorted from B16F10-OVA tumors. **(C)** Expression of FRβ in wild-type and *Folr2*^*−/−*^ BMDMs and TAMs isolated from B16F10 tumors. **(D)** Tumor growth data for the indicated tumor cell lines implanted into wild-type or *Folr2*^*−/−*^ mice, pooled from 3 experiments (B16F10), 5 experiments (B16F10-OVA), 2 experiments (MCA205), and 4 experiments (MC38). **(E)** scRNA-seq profiles of tumor-infiltrating myeloid and dendritic cells from B16F10 tumors implanted into wild-type or *Folr2*^*−/−*^ mice. UMAP of major myeloid clusters shown. **(F)** Cell density via galaxy plot in UMAP space (left) and quantification of the percentage of cells in each cluster between wild-type and *Folr2*^*−/−*^ mice (right). Pooled from 5 tumors implanted in each genotype of mice. **(G)** Expression of key genes in individual myeloid cells from B16F10 tumors. **(H)** Gene set enrichment analysis (GSEA) of indicated signatures in the ranked list of genes differentially expressed between all tumor-infiltrating myeloid cells (dendritic cells excluded) isolated from wild-type and *Folr2*^*−/−*^ mice. **(I)** Flow cytometry analysis of wild-type and *Folr2*^*−/−*^ TAMs isolated from B16F10 tumors stained for CD86, MHC-II, CD206, and Arg1. Representative flow plots and pooled bar plots from 2 independent experiments shown. **(J)** Frequency of IL-12/23p40+ TAMs isolated from wild-type or *Folr2*^*−/−*^ mice and cultured for 18h *ex vivo*. Representative contour flow plots (left) and quantification (right) pooled between three independent experiments. **(K)** 1×10^6^ wild-type or *Folr2*^*−/−*^ BMDMs cultured in low folate media without polarizing cytokines were injected intratumorally on day 9 into established B16F10-OVA tumors implanted into wild-type mice. Tumor growth measured. One of 3 independent experiments shown. * p < 0.05; ** p < 0.01; *** p < 0.001; **** p < 0.0001; ns not significant. Created in BioRender. Zimmerman, M. (2027) https://BioRender.com/kn294bd, https://BioRender.com/3tahq1f

**Figure 3:**
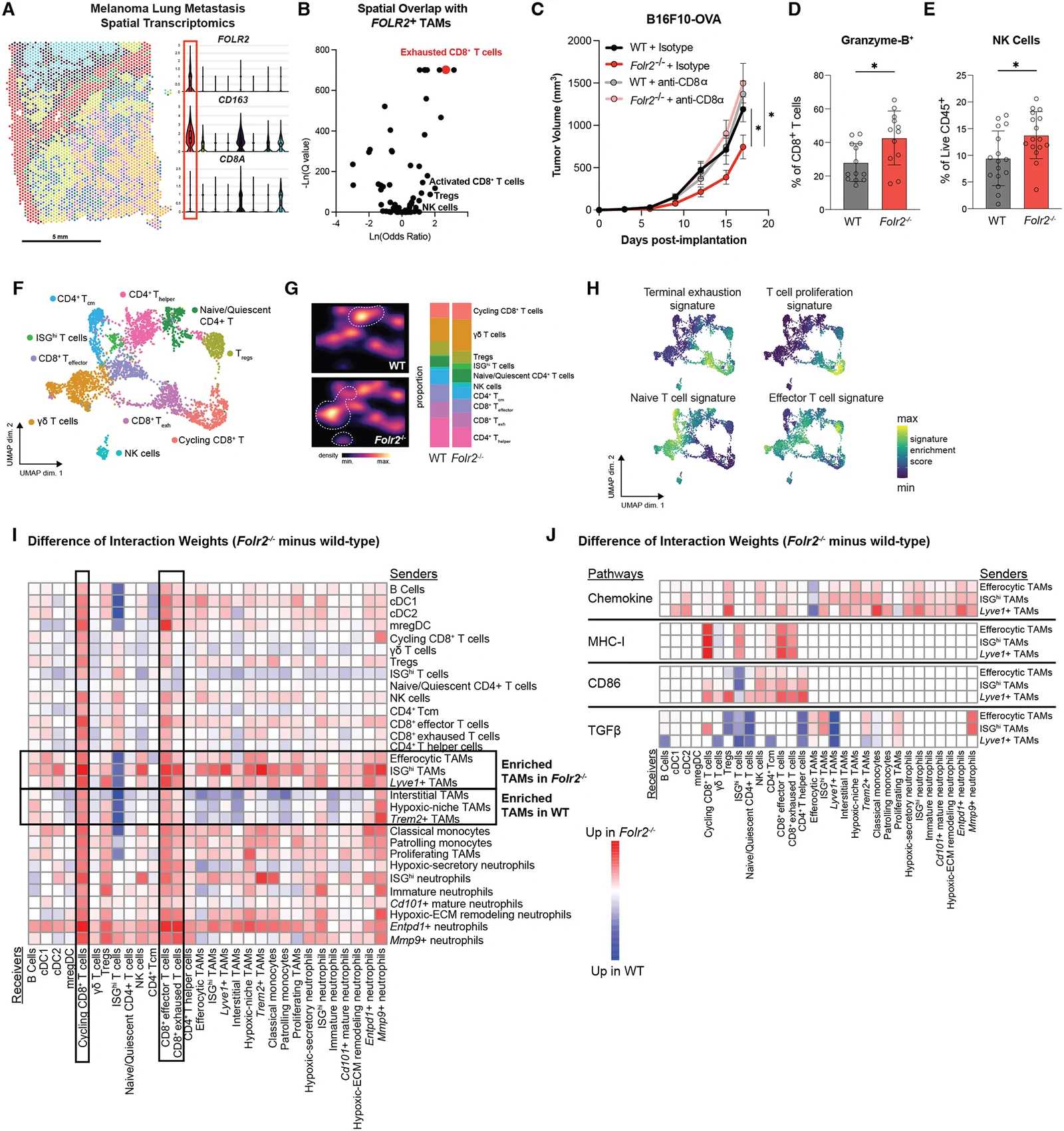
*Folr2*^*−/−*^ TAMs promote tumor-infiltrating lymphocyte activation and anti-tumor immunity. **(A)** Visium spatial transcriptomic analysis of a melanoma lung metastasis sample, demonstrating a cluster containing *FOLR2+* TAMs and few CD8^+^ T cells (red). **(B)** Volcano plot of spatial overlap (Fisher’s exact test) of *FOLR2+* TAMs with different immune cells from 22 melanoma resections across 4 metastatic sites. **(C)** B16F10-OVA tumor volumes from wild-type or *Folr2*^*−/−*^ mice treated with anti-CD8α depleting or isotype control antibodies. Results pooled from three independent experiments. **(D)** Quantification of Granzyme B-expressing CD8^+^ T cells from B16F10-OVA tumors. **(E)** Quantification of NK cells in B16F10 tumors in wild-type and *Folr2*^*−/−*^ mice. **(F)** scRNA-seq profiles of tumor-infiltrating lymphocytes cells from B16F10 tumors implanted into wild-type or *Folr2*^*−/−*^ mice. UMAP of major lymphocyte clusters shown. **(G)** Cell density via galaxy plot in UMAP space (left) and quantification of the percentage of cells in each cluster between wild-type and *Folr2*^*−/−*^ mice (right). Pooled from 5 tumors implanted in each genotype of mice. **(H)** Expression of indicated signatures in lymphocytes from B16F10 tumors. **(I)** Heatmap showing difference of weighted interactions (*Folr2*^*−/−*^ minus wild-type) between single-cell transcriptional clusters. Positive scores (red) indicate higher interaction between cells from *Folr2*^*−/−*^ mice, negative scores (blue) indicate higher interaction between cells from wild-type mice. **(J)** Heatmap showing difference of weighted interactions (*Folr2*^*−/−*^ minus wild-type) for the specific pathways and sender populations shown. * p < 0.05; ** p < 0.01; *** p < 0.001; **** p < 0.0001; ns not significant.

**Figure 4:**
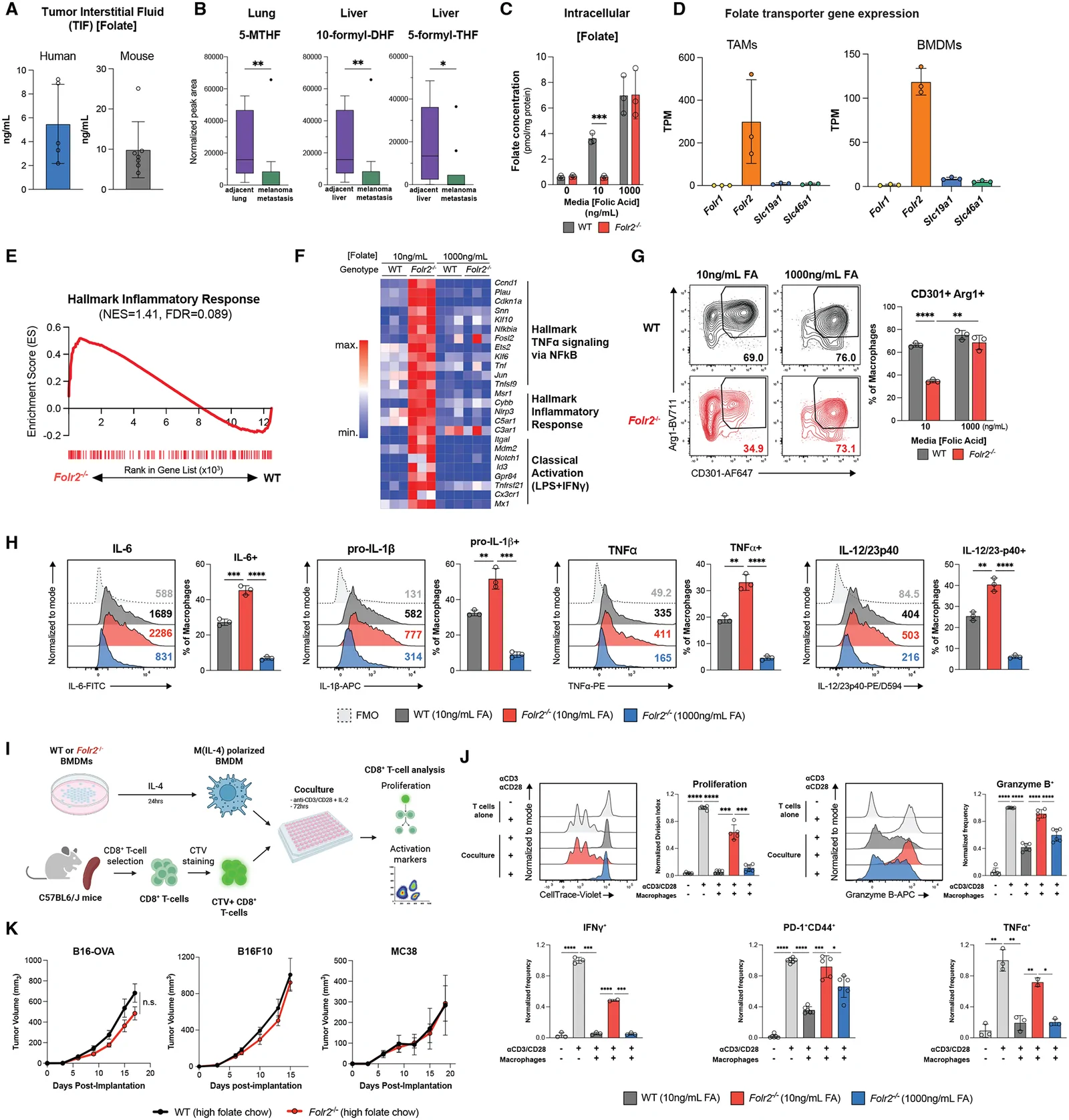
Folate uptake by FRβ is required for immunosuppressive macrophage polarization. **(A)** Measurement of tumor interstitial fluid (TIF) from human and mouse tumors by folate ELISA. **(B)** Untargeted metabolomics performed on human metastatic melanoma samples and adjacent normal lung and liver. Folate metabolites shown. **(C)** Intracellular folate measurements by ELISA of BMDMs grown for 6 days in folate-free media supplemented with 0, 10, or 1000ng/mL folic acid normalized to protein quantification. **(D)** Folate receptor and transporter expression in RNA-seq data from TAMs flow-sorted from B16-ova tumors or M(IL-4) BMDMs grown in low folate media. Transcripts per million (TPM) shown. **(E)** GSEA of Hallmark Inflammatory Response signature in the ranked list of genes differentially expressed between wild-type and *Folr2*^*−/−*^ M(IL-4) BMDMs grown in low folate media. **(F)** Heatmap of leading edge genes from pro-inflammatory signatures showing upregulation in *Folr2*^*−/−*^ M(IL-4) BMDMs cultured in low folate media, but not high folate media. **(G)** Representative flow plots (left) and quantification (right) of CD301 and Arg1 expression in wild-type and *Folr2*^*−/−*^ M(IL-4) BMDMs. 1 of 2 independent experiments shown. **(H)** Representative histograms and quantification of intracellular cytokine staining in wild-type and *Folr2*^*−/−*^ M(IL-4) BMDMs. 1 of 3 independent experiments shown. **(I)** Schema of *in vitro* CD8^+^ T cell inhibition assay with BMDMs. **(J)** Flow plots and quantification of CellTrace Violet dilution and expression of Granzyme B, PD-1^+^CD44^+^, IFNу, and TNFα by flow cytometry. Pooled data from two of three or data from one of three independent experiments shown. Data normalized to stimulated T cell condition without macrophages. **(K)** Tumor growth curves of B16F10-OVA, B16F10, and MC38 in wild-type and *Folr2*^*−/−*^ fed high folate diet for 4 weeks prior to tumor implantation. Data pooled from 1–3 independent experiments. * p < 0.05; ** p < 0.01; *** p < 0.001; **** p < 0.0001; ns not significant. Created in BioRender. Zimmerman, M. (2027) https://BioRender.com/a4m6ekl

**Figure 5:**
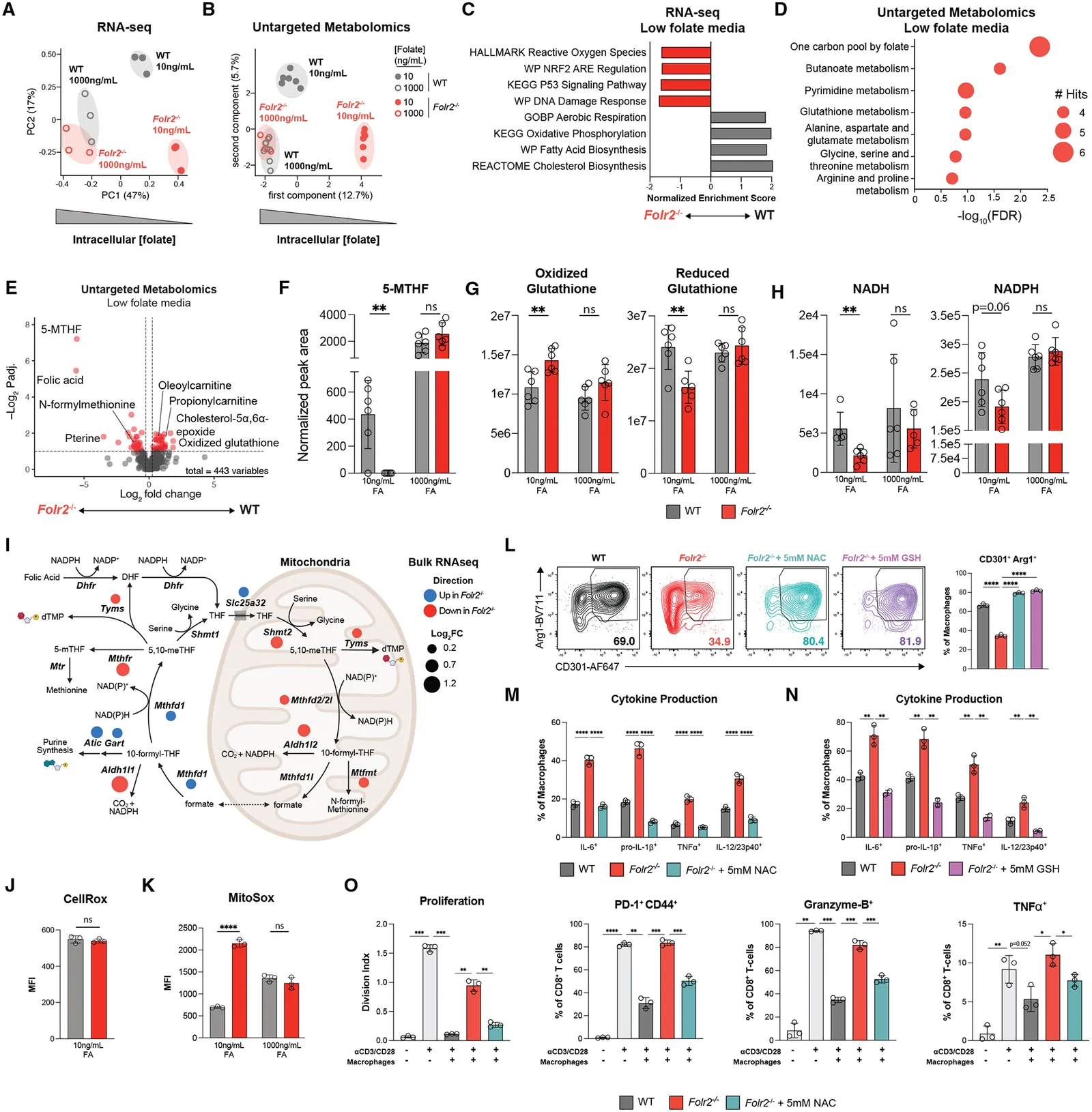
Folate deficiency induces oxidative stress in *Folr2*^*−/−*^ macrophages through dysregulation of glutathione metabolism. **(A)** PCA of RNA-seq and **(B)** PLS-DA of untargeted metabolomics data from wild-type and *Folr2*^*−/−*^ M(IL-4) BMDMs grown in low folate or high folate media. **(C)** Normalized enrichment scores of indicated signatures comparing wild-type and *Folr2*^*−/−*^ M(IL-4) BMDMs grown in low folate media. **(D)** Metabolic pathways enriched in the comparison between wild-type and *Folr2*^*−/−*^ BMDMs grown in low folate media. Size of dots represents the number of significant differentially detected metabolites in each pathway. **(E)** Volcano plot of differentially detected metabolites comparing wild-type and *Folr2*^*−/−*^ M(IL-4) BMDMs grown in low folate media. Cutoffs set at absolute Log_2_FC > 0.25, FDR < 0.1. **(F-H)** Normalized peak area of 5-methyltetrahydrofolate (5-MTHF) **(F)**, reduced and oxidized glutathione **(G)**, and NADH/NADPH **(H)** detected in wild-type and *Folr2*^*−/−*^ BMDMs grown in low or high folate media. **(I)** Schema of cytosolic and mitochondrial 1C metabolic pathway, with enzymes identified as upregulated (blue) or downregulated (red) in *Folr2*^*−/−*^ BMDMs compared with wild-type BMDMs. Size of the colored dots represents Log_2_FC. Enzymes without dots are not significantly different. **(J, K)** Representative flow plots and quantification of CellRox **(J)** or MitoSox **(K)** staining of wild-type and *Folr2*^*−/−*^ BMDMs grown in low or high folate media. **(L-N)** Quantification of CD301 and Arg1 expression **(H)** or intracellular cytokine production **(I)** in wild-type and *Folr2*^*−/−*^ M(IL-4) BMDMs grown in low folate media +/− 5mM glutathione (GSH) or 5mM N-acetylcysteine (NAC). One of two independent experiments shown. **(O)** Quantification of T cell proliferation by CellTrace Violet, intracellular cytokine production (IFNу, TNFα), and T cell activation markers (Granzyme B, PD-1^+^CD44^+^ expression) after co-culture of CD8^+^ T cells with wild-type or *Folr2*^*−/−*^ M(IL-4) BMDMs grown in low folate media +/− 5mM N-acetylcysteine (NAC). One of two independent experiments shown. * p < 0.05; ** p < 0.01; *** p < 0.001; **** p < 0.0001; ns not significant. BioRender license: Created in BioRender. Zimmerman, M. (2027) https://BioRender.com/8178nij

**Figure 6:**
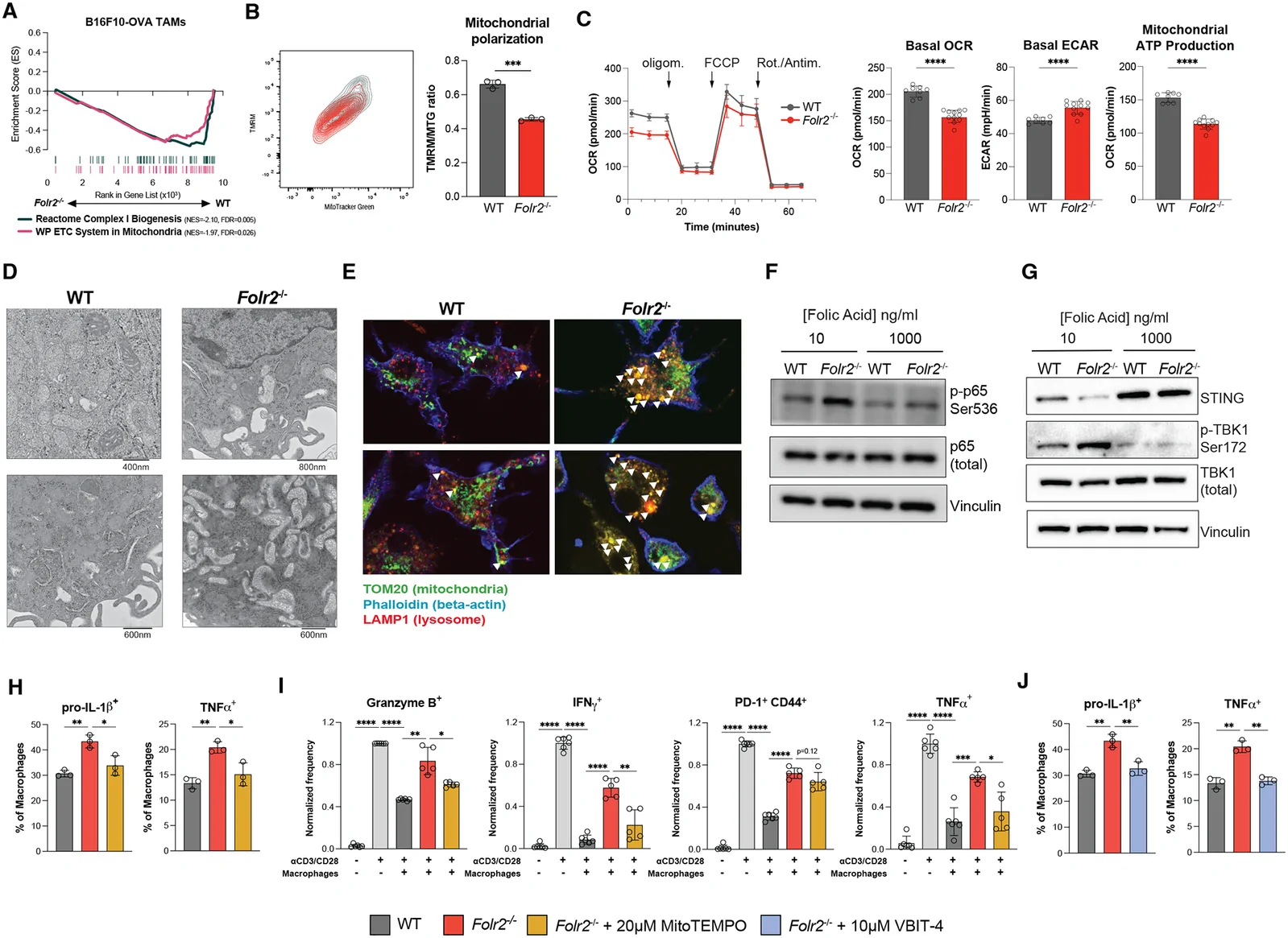
Dysfunctional mitochondria promote pro-inflammatory polarization of *Folr2*^*−/−*^ macrophages. **(A)** GSEA of mitochondrial gene signatures in the ranked list of genes differentially expressed between wild-type and *Folr2*^*−/−*^ TAMs flow-sorted from B16F10-OVA tumors. **(B)** Tetramethylrhodamine methyl ester (TMRM) and Mitotracker Green staining of wild-type and *Folr2*^*−/−*^ M(IL-4) BMDMs grown in low folate media. Representative flow plot (left) and quantification of one of three representative experiments (right) shown. **(C)** Seahorse XF Mito Stress Test of wild-type and *Folr2*^*−/−*^ M(IL-4) BMDMs grown in low folate media. Representative curve (left) and bar plots of key measurements (right) - basal oxygen consumption rate (OCR), basal extracellular acidification rate (ECAR), and mitochondrial ATP production - shown from one of three independent experiments. **(D)** Representative transmission electron microscopy images of wild-type and *Folr2*^*−/−*^ M(IL-4) BMDMs grown in low folate media showing mitochondria and vesicles containing mitochondrial remnants. **(E)** Immunofluorescence images of wild-type and *Folr2*^*−/−*^ M(IL-4) BMDMs grown in low folate media stained with antibodies against LAMP1 (to identify lysosomes) and TOM20 (mitochondria), as well as Phalloidin (β-actin). Mitochondria in phagolysosomes identified by overlapping staining for LAMP1 and TOM20 (white arrowhead). **(F-G)** Immunoblot of lysates from wild-type and *Folr2*^*−/−*^ M(IL-4) BMDMs grown in low or high folate media stained against phospho-p65, total p65, STING, phospho-TBK1, total TBK1, and vinculin. **(H, J)** Quantification of intracellular cytokine production in wild-type and *Folr2*^*−/−*^ M(IL-4) BMDMs grown in low folate media +/− 20μM MitoTEMPO or 10μM VBIT-4. One of two independent experiments shown. **(I)** Quantification of T cell intracellular cytokine production (IFNу, TNFα), and T cell activation markers (Granzyme B, PD-1^+^CD44^+^ expression) after co-culture of CD8^+^ T cells with wild-type or *Folr2*^*−/−*^ M(IL-4) BMDMs grown in low folate media +/− 20μM MitoTEMPO. One of two independent experiments shown. * p < 0.05; ** p < 0.01; *** p < 0.001; **** p < 0.0001; ns not significant.

**Figure 7:**
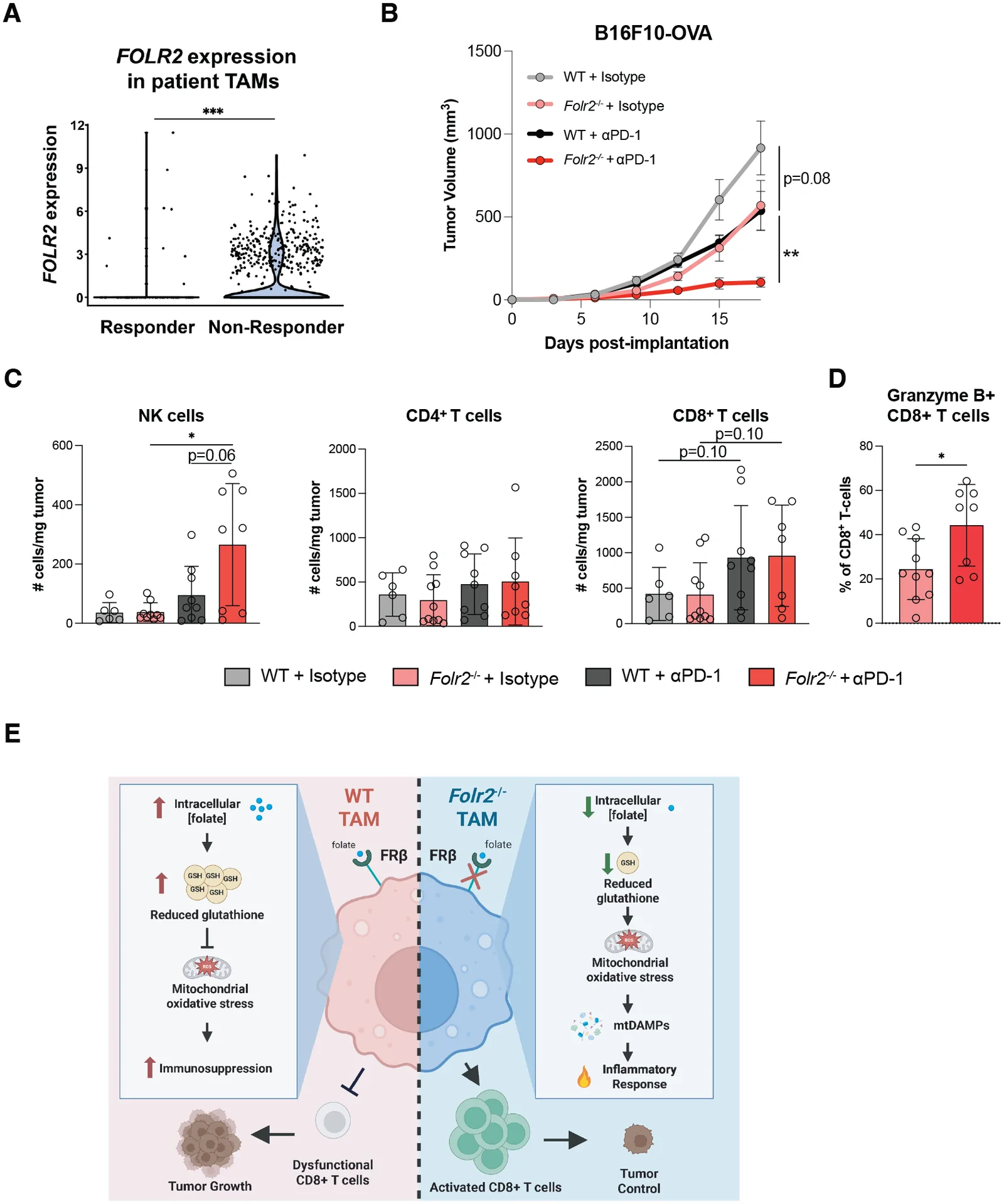
Loss of *Folr2* increases the efficacy of immune checkpoint inhibitor therapy. **(A)** ScRNA-seq of *FOLR2* expression in TAMs from ICI-responder and non-responder melanoma patients from Sade-Feldman *et al*. **(B)** Growth curves of B16F10-OVA tumors implanted into wild-type or *Folr2*^*−/−*^ mice treated with either anti-PD-1 (αPD-1) or isotype control antibodies every 3 days starting once tumors reached an average of 100mm^3^. Pooled results from two independent experiments. **(C)** Quantification of NK, CD4^+^, and CD8^+^ T cell infiltration into tumors from the four treatment conditions shown in **(B)**. **(D)** Quantification of Granzyme B-expressing CD8^+^ T cells in *Folr2*^*−/−*^ mice. **(E)** Schema of proposed mechanism for the role of FRβ and folate metabolism in TAMs. * p < 0.05; ** p < 0.01; *** p < 0.001; **** p < 0.0001; ns not significant. Created in BioRender. Zimmerman, M. (2027) https://BioRender.com/9c5ut7w; https://BioRender.com/a508l9s
